# Supplementary material for: Synthesis and Anticancer Activity of Novel Dual Inhibitors of Human Protein Kinases CK2 and PIM-1
Source: Pharmaceutics. 2023 Jul 20;15(7):1991. doi: 10.3390/pharmaceutics15071991 (PMC10385865; doi:10.3390/pharmaceutics15071991)
Supplement: Supplementary file 1 [file pharmaceutics-15-01991-s001.zip › pharmaceutics-2445054-supplementary.pdf]

Supporting Information

for

Synthesis and Anti-Cancer Activity of Novel Dual Inhibitors of Human Protein Kinases CK2 and PIM-1

Patrycja Wińska,*[,a] Monika Wielechowska,[a]
Mirosława Koronkiewicz,[b] and Paweł Borowiecki*[,a]

Addresses: [a] Faculty of Chemistry, Warsaw University of Technology, Noakowskiego 3, Warsaw 00–664, Poland. [b] National Medicines Institute, Chełmska 30/34, Warsaw 00–725, Poland.

Email addresses of corresponding authors:
Dr. Patrycja Wińska – patrycja.winska@pw.edu.pl
Dr. Paweł Borowiecki – pawel.borowiecki@pw.edu.pl

Table of contents

1. General information …………………………………………………..…..……………... S2

2. Synthetic procedures for the obtained compounds ………..…………...……………. S3–S8

3. Biological assays ……………………………………………….......................... S9–S11

4. Evaluation of ADMET profile for the obtained compounds ………….…………..… S12–S16

5. Molecular docking ……………….……………………………......................... S17–S21

6. Analytical data (copies of HPLC chromatograms) ………………............……... S22–S23

7. Spectral data (copies of NMR and FTMS spectra) ……………........................... S24–S36

8. References ……..………………………………………………..…………………..….. S37

1. General information.

Chromatography grade *n*-hexane and 2-propanol (2-PrOH) used in high-performance liquid chromatography (HPLC) were purchased from Avantor Performance Materials Poland S.A. (formerly POCH Polish Chemicals Reagents). All other commercially available reagents [purchased from Merck KGaA, (Darmstadt, Germany), TCI (Tokyo Chemical Industry), Thermo Fisher (Kandel) GmbH (Kandel, Germany), and Fluorochem Ltd. (Hadfield Derbyshire, United Kingdom)] were used without further purification.

Analytical thin-layer chromatography was carried out on TLC aluminium plates with silica gel Kieselgel 60 F_{254} (Merck, Germany) (0.2 mm thickness film containing a fluorescence indicator green 254 nm (F254) using UV light as a visualizing agent.

The gas chromatography (GC) analyses were performed with an Agilent Technologies 6890N instrument (Maryland, United States) equipped with a flame ionization detector (FID) and fitted with HP-50+ (30 m) semi-polar column (50 % phenyl–50 % methylpolysiloxane); the GC injector was maintained at 250 °C; Helium (2 mL/min) was used as carrier gas; retention times (t_R) are given in minutes under these conditions.

The enantiomeric excesses (% ee) of biocatalytic reduction products were determined by high-performance liquid chromatography (HPLC) analyses performed on Shimadzu Nexera-*i* (LC-2040C 3D) equipped with a photodiode array detector (PAD) using Chiralpak AD-H (4.6 mm × 250 mm, coated on 5 µm silica gel grain size, from Daicel Chemical Ind., Ltd., Japan) as a chiral column; the respective mixture of *n*-hexane/2-PrOH was used as mobile phases in the appropriate ratios; the HPLC analyses were executed in an isocratic and isothermal (25 °C) mode; flow (*f*) is given in mL/min.

^{1}H NMR (500 MHz), ^{13}C NMR (126 MHz), and ^{19}F NMR (470 MHz) spectra were recorded on a Varian NMR System 500 MHz spectrometer; The ^{1}H, ^{13}C, and ^{19}F chemical shifts (δ) are reported in parts per million (ppm) relative to the (i) solvent signals {$CDCl_3$, δ_H (residual $CHCl_3$) 7.26 ppm, δ_C 77.16 ppm or DMSO-d_6, δ_H [residual $(CD_3)_2SO$] 2.49 ppm with HDO at 3.30 ppm, δ_C 40.45 ppm or acetone-d_6, δ_H [residual $(CD_3)_2CO$] 2.05 ppm with HDO at 2.81 ppm, δ_C 206.26 and 29.84 ppm} or internal $CFCl_3$ reference set at 0 ppm. Attention: ^{19}F NMR (470 MHz) spectra was recorded in deuterated acetonitrile (CD_3CN). Chemical shifts are quoted as s (singlet), d (doublet), dd (doublet of doublets), t (triplet), q (quartet), m (multiplet), and br. s (broad singlet); coupling constants (*J*) are reported in Hertz (Hz). Raw spectroscopic data have been processed with MestReNova (Product version: 6.0.2-5475).

Fourier Transform Mass Spectrometry (FTMS) spectra were recorded on Q Exactive Hybrid Quadrupole-Orbitrap Mass Spectrometer, ESI source: electrospray with spray voltage 4.00 kV; all samples were prepared by dilution with MeOH (0.5 mL) and addition of a mixture of CH_3CN/MeOH/H_2O (50:25:25, v/v/v) + 0.5% formic acid (HCOOH) each.

2. Synthetic procedures for the obtained compounds.

2.1. General procedure for the synthesis of 2-bromo-1H-benzimidazole (2)

Compound **2** was synthesized according to procedure reported by Ellingboe et al. [1]. To a cooled (water bath, 5–10 °C), mechanically stirred mixture of 2-mercapto-1*H*-benzimidazole (**1**, 10.0 g, 66.58 mmol), 48% aqueous HBr (10 mL), and glacial AcOH (100 mL), Br_2 (12 mL, 0.24 mol) was added dropwise over 25 min. The mixture warmed slightly (40–45 °C) during the addition, and additional glacial AcOH (50 mL) was added to aid the stirring of the thick mixture. After the addition was complete, stirring was continued at room temperature for 4 h. Afterwards, H_2O (200 mL) was added, and the resulting solution was cooled in an ice bath (0–5 °C). The pH was adjusted to 4 with solid NaOH (ca. 40 g), and the precipitate was collected by filtration to give crude product which was further purified by recrystallization from acetone to afford 2-bromo-1*H*-benzimidazole (**2**, 6.93 g, 35.17 mmol, 53% yield) as white solid.

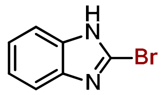

2

Yield 53% (6.93 g); white solid; mp 194–196 °C (Acetone) [1] 190–192 °C (Acetone)]; R_f [CH_2Cl_2/MeOH (99:1, v/v)] 0.24; ^1H NMR (500 MHz, DMSO-d_6): δ 13.18 (br. s, 1H), 7.51 (s, 2H), 7.19 (m, 2H), 2.07 (s, 1H); ^{13}C NMR (126 MHz, DMSO-d_6): δ 139.7, 127.2, 122.2, 114.6; MS (ESI-TOF) *m/z*: $[M+H]^+$ Calcd for $C_7H_6BrN_2^+$ m/z: 196.9709, Found 196.9697; FTMS (ESI-TOF) *m/z*: $[M+H]^+$ Calcd for $C_7H_6BrN_2^+$ m/z: 196.97089 and 198.96884, Found 196.97125 and 198.96854; GC [200–260 (10 °C/min)]: t_R=3.16 min.

2.2. General procedure for the synthesis of 2,4,5,6,7-pentabromo-1H-benzimidazole (3)

Compound **3** was synthesized according to procedure reported by Kazimierczuk et al. [2]. To a stirred and refluxed suspension of 2-bromo-1*H*-benzimidazole (**2**, 1.5 g, 7.6 mmol) in H_2O (60 mL), Br_2 (8 mL, 160 mmol) was added portionwise within 6 h. The reflux was continued for 24 h under irradiation of purple LEDs (390 nm) (For details see **Figure S1** below). Afterwards, the reaction mixture was cooled, and the orange precipitate was filtered off under suction. The collected solid was dissolved in MeOH/25% $NH_{3aq.}$ (80 mL, 3:1, v/v), treated with charcoal (1.3 g) and cellite (3.5 g). After filtering of the solids under suction, the pale-yellow solution of the permeate was brought to pH 4–5 with conc. AcOH (150 mL) and the formed precipitate was recrystallized from MeOH/H_2O (50 mL, 1:1, v/v) to give 2,4,5,6,7-pentabromo-1*H*-benzimidazole (**3**, 1.65 g, 3.22 mmol, 42%) as yellowish solid.

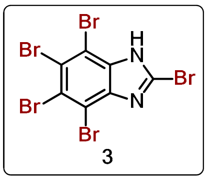

Yield 42% (1.65 g); yellowish solid; R_f [hexane/AcOEt (50:50, v/v)] 0.15; ^{1}H NMR (500 MHz, DMSO-d_6): δ Not found; ^{13}C NMR (126 MHz, DMSO-d_6 + 2 drops of 2M HCl$_{aq.}$ and registered for 16 h): δ 142.7, 138.9, 138.0, 131.3, 121.0, 120.9, 110.3; FTMS (ESI-TOF) *m/z*: [M+H]$^+$ Calcd for $C_7H_2Br_5N_2^+$ m/z: 512.60884, Found 512.60913.

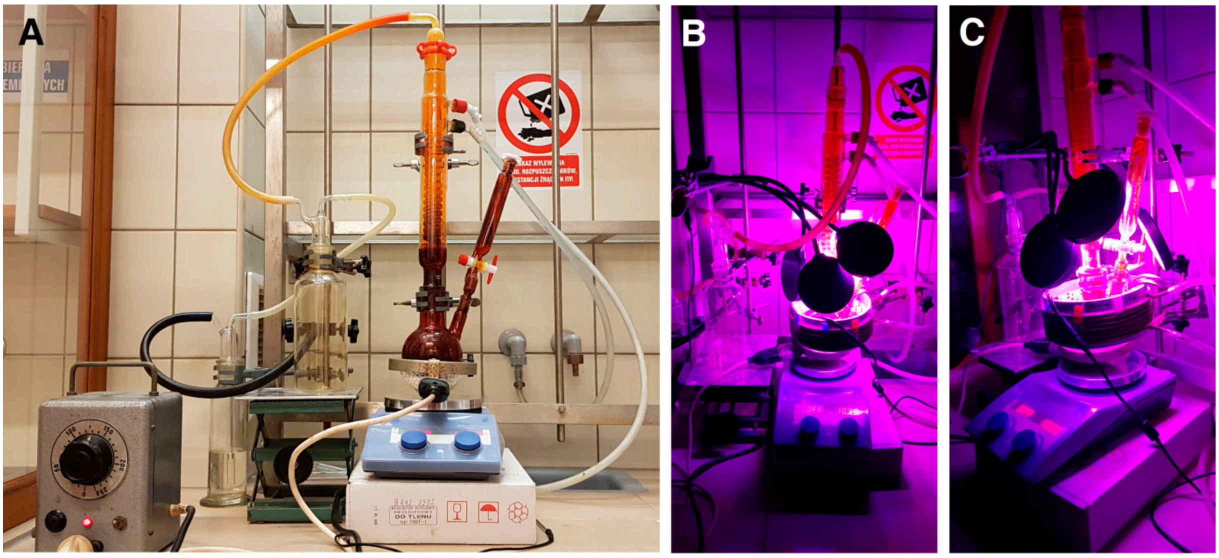

Figure S1. Synthesis of 2,4,5,6,7-pentabromo-1*H*-benzimidazole (**3**) as a key precursor for novel dual CK2/PIM-1 inhibitors carried out under (**A**) convenient heating conditions, and (**B**–**C**) the influence of purple light (390 nm). Light-induced chemical reactions was conducted using self-prepared (made-in-home) device equipped with light-emitting diodes (LEDs).

2.3. General procedure for the synthesis of dual CK2/PIM-1 inhibitors - TBBi amino alcohol derivatives (4–11)

Compounds **4**–**11** were synthesized according to procedure reported by Kazimierczuk and Pinna et al. [3]

Method A: A mixture of 2,4,5,6,7-pentabromo-1*H*-benzimidazole (**3**, 150 mg, 0.30 mmol) and the respective amino alcohol (4.9 equiv) in anhydrous EtOH (4.5 mL) was heated in an ace pressure tube (bushing type, Back seal, V = 15 mL, L × O.D. 10.2 cm × 25.4 mm, Sigma Aldrich: Z181064) at 110–115 °C for 72 h. Afterwards, the reaction mixture was cooled to room temperature, and the volatiles were evaporated under reduced pressure using rotavap. The oil residue was purified by column chromatography packed with SiO_2 using a sequential mixture of hexane/AcOEt (500 mL, 50:50 v/v) and $CHCl_3$/MeOH (500 mL, 90:10 v/v) as an eluent to afford desired solid-state product **4**, *rac*-**6**, **8**, *rac*-**9**, **10**, and **11**.

Method B: A mixture of 2,4,5,6,7-pentabromo-1*H*-benzimidazole (**3**, 500 mg, 0.98 mmol) and racemic 2-hydroxypropylamine (1.10 g, 14.63 mmol, 1.13 mL) in anhydrous EtOH (7.5 mL) was heated in an ace pressure tube (bushing type, Back seal, V = 15 mL, L × O.D. 10.2 cm × 25.4 mm, Sigma Aldrich: Z181064) at 110–115 °C for 72 h. Afterwards, the reaction mixture was cooled to room temperature, and the volatiles were evaporated under reduced pressure using rotavap. The oil residue was purified by column chromatography packed with SiO_2 using a sequential mixture of hexane/AcOEt (500 mL, 50:50 v/v) and $CHCl_3$/MeOH (500 mL, 90:10 v/v) as an eluent to afford desired product 1-[(4,5,6,7-tetrabromo-1*H*-benzimidazol-2-yl)amino]propan-2-ol (*rac*-**5**, 247 mg, 0.49 mmol, 50%) as white solid.

Method C: A mixture of 2,4,5,6,7-pentabromo-1*H*-benzimidazole (**3**, 50 mg, 98 μmol) and optically active (*S*)- or (*R*)-1-aminopropan-2-ol (110 mg, 1.46 mmol, 113 μL) in dry $PhCH_3$ (1 mL) was heated in an ace pressure tube (bushing type, Back seal, V = 15 mL, L × O.D. 10.2 cm × 25.4 mm, Sigma Aldrich: Z181064) at 100 °C for 48 h. Afterwards, the reaction mixture was cooled to room temperature, and the volatiles were evaporated under reduced pressure using rotavap. The oil residue was purified by column chromatography packed with SiO_2 using $CHCl_3$/MeOH (95:5 v/v) as an eluent to afford desired enantiomerically pure (2*S*)-1-[(4,5,6,7-tetrabromo-1*H*-benzimidazol-2-yl)amino]propan-2-ol [(*S*)-**5**, 46 mg, 91 μmol, 93% yield, >99% ee] or (2*R*)-1-[(4,5,6,7-tetrabromo-1*H*-benzimidazol-2-yl)amino]propan-2-ol [(*R*)-**5**, 42 mg, 83 μmol, 85% yield, >99% ee] as white solid, respectively.

4,5,6,7-Tetrabromo-N,N-dimethyl-1H-benzimidazol-2-amine (DMAT, 4)

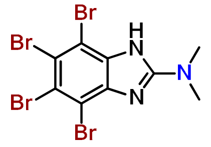

DMAT (4)

Synthesized according to *Method A* (section: 2.3). Yield 29% (41 mg); white solid; R_f [$CHCl_3$/MeOH (95:5 v/v)] 0.82; ^1H NMR (500 MHz, DMSO-d_6): δ 11.44 (br. s, 1H, N*H*), 3.13 (s, 6H, C*H*3); ^{13}C NMR (126 MHz, DMSO-d_6): δ 158.1 (*C*NCH3), 38.3 (*C*H3), the rest of the peaks were not detected; FTMS (ESI-TOF) *m/z*: $[M+H]^+$ Calcd for $C_9H_8Br_4N_3^+$ m/z: 477.74053, Found 477.74015.

1-[(4,5,6,7-Tetrabromo-1H-benzimidazol-2-yl)amino]propan-2-ol (rac-5)

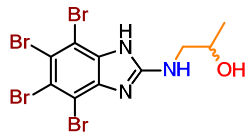

rac-5

Synthesized according to *Method B* (section: 2.3). Yield 50% (247 mg); white solid; R_f [hexane/AcOEt (50:50, v/v)] 0.22 or R_f [$CHCl_3$/MeOH (90:10, v/v)] 0.60 or R_f [$CHCl_3$/MeOH (95:5 v/v)] 0.54; ^1H NMR (500 MHz, acetone-d_6): δ 11.04 (br. s, 1H), 6.48 (br. s, 1H), 4.70 (br. s, 1H),

4.07–3.92 (m, 1H), 3.56 (ddd, J=13.6, 6.5, 3.5 Hz, 1H), 3.33 (ddd, J=13.5, 7.3, 5.1 Hz, 1H), 1.19 (d, J=6.3 Hz, 3H); ^{1}H NMR (500 MHz, DMSO-d_6): δ 11.41 (br. s, 1H), 6.62 (br. s, 1H), 4.95 (br. s, 1H), 3.83 (d, J=5.9 Hz, 1H), 3.38 (ddd, J=13.2, 6.5, 4.4 Hz, 1H), 3.26–3.18 (m, 1H), 1.10 (d, J=6.2 Hz, 3H); ^{13}C NMR (126 MHz, DMSO-d_6 + 2 drops of 2M $HCl_{aq.}$ and registered for 16 h): δ 154.8, 153.2, 131.5, 130.5, 120.8, 118.0, 106.4, 103.7, 65.2, 50.1, 20.8; FTMS (ESI-TOF) *m/z*: $[M+H]^+$ Calcd for $C_{10}H_{10}Br_4N_3O^+$ m/z: 507.75110, Found 507.75086; HPLC [*n*-hexane-2-PrOH (95:5, v/v); f=0.8 mL/min; λ=225 nm; T=25 °C (Chiralpak AD-H)]: t_R = 19.105 (*S*-isomer) and 21.524 min (*R*-isomer).

1,1,1-Trifluoro-3-[(4,5,6,7-tetrabromo-1H-benzimidazol-2-yl)amino]propan-2-ol (rac-6)

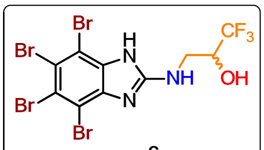

rac-6

Synthesized according to *Method A* (section: 2.3). Yield 28% (46 mg); beige solid; R_f [$CHCl_3$/MeOH (95:5 v/v)] 0.55; ^{1}H NMR (500 MHz, DMSO-d_6): δ 11.69 (br. s, 1H), 6.76 (t, J=5.9 Hz, 1H), 6.66 (br. s, 1H), 4.39–4.27 (m, 1H), 3.70 (ddd, J=13.8, 6.1, 4.0 Hz, 1H), 3.49 (ddd, J = 13.9, 8.1, 5.8 Hz, 1H); ^{19}F NMR (470 MHz, CD_3CN): δ –77.26 (d, J =7.4 Hz, 3F).; FTMS (ESI-TOF) *m/z*: $[M+H]^+$ Calcd for $C_{10}H_7Br_4F_3N_3O^+$ m/z: 561.72283, Found 561.72251.

3-[(4,5,6,7-Tetrabromo-1H-benzimidazol-2-yl)amino]propan-1-ol (7)

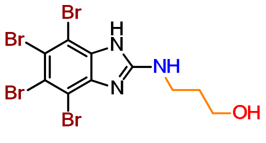

7

Synthesized according to *Method A* (section: 2.3). Yield 44% (67 mg); light green solid; R_f [$CHCl_3$/MeOH (95:5 v/v)] 0.32; ^{1}H NMR (500 MHz, DMSO-d_6): δ 11.59 (br. s, 1H), 6.77 (t, J=5.4 Hz, 1H), 3.50 (t, J=6.1 Hz, 4H), 3.43 (q, J=6.6 Hz, 2H), 1.71 (p, J=6.5 Hz, 2H); FTMS (ESI-TOF) *m/z*: $[M+H]^+$ Calcd for $C_{10}H_{10}Br_4N_3O^+$ m/z: 507.75110, Found 507.75116.

2-[Methyl(4,5,6,7-tetrabromo-1H-benzimidazol-2-yl)amino]ethanol (8)

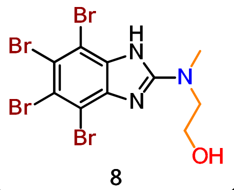

8

Synthesized according to *Method A* (section: 2.3). Yield 37% (56 mg); white solid; R_f [$CHCl_3$/MeOH (95:5 v/v)] 0.60; ^{1}H NMR (500 MHz, DMSO-d_6): δ 11.44 (br. s, 1H), 4.89 (br. s, 1H), 3.67–3.59 (m, 4H), 3.17 (s, 3H); FTMS (ESI-TOF) *m/z*: $[M+H]^+$ Calcd for $C_{10}H_{10}Br_4N_3O^+$ m/z: 507.75110, Found 507.75116.

1-(Diethylamino)-3-[(4,5,6,7-tetrabromo-1H-benzimidazol-2-yl)amino]propan-2-ol (rac-9)

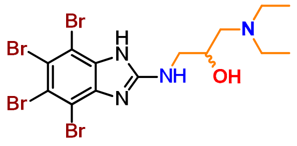

rac-9

Synthesized according to *Method A* (section: 2.3). Yield 64% (112 mg); white solid; R_f [$CHCl_3$/MeOH (95:5 v/v)] 0.48; ^1H NMR (500 MHz, DMSO-d_6): δ 8.22 (br. s, 1H), 5.32 (dt, J=11.1, 5.5 Hz, 1H), 5.04 (ddd, J=13.3, 5.8, 4.4 Hz, 1H), 4.85–4.76 (m, 1H), 4.24–3.80 (m, 12H); FTMS (ESI-TOF) *m/z*: $[M+H]^+$ Calcd for $C_{14}H_{19}Br_4N_4O^+$ m/z: 578.82459, Found 578.82454.

2-[(4,5,6,7-Tetrabromo-1H-benzimidazol-2-yl)amino]propane-1,3-diol (10)

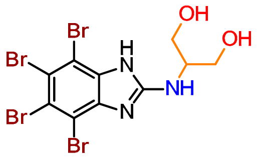

10

Synthesized according to *Method A* (section: 2.3). Yield 13% (20 mg); white solid; R_f [$CHCl_3$/MeOH (95:5 v/v)] 0.10; ^1H NMR (500 MHz, DMSO-d_6): δ 6.39 (d, J=7.7 Hz, 1H), 3.88–3.79 (m, 1H), 3.55 (qd, J=10.7, 5.4 Hz, 4H); FTMS (ESI-TOF) *m/z*: $[M+H]^+$ Calcd for $C_{10}H_{10}Br_4N_3O_2^+$ m/z: 523.74601, Found 523.74597.

3-[(4,5,6,7-Tetrabromo-1H-benzimidazol-2-yl)amino]propane-1,2-diol (rac-11)

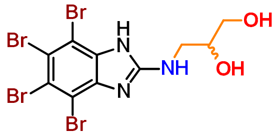

rac-11

Synthesized according to *Method A* (section: 2.3). Yield 38% (60 mg); white solid; R_f [$CHCl_3$/MeOH (95:5 v/v)] 0.13; ^1H NMR (500 MHz, DMSO-d_6): δ 6.61 (t, J=5.3 Hz, 1H), 4.09 (d, J=4.7 Hz, 1H), 3.70–3.60 (m, 1H), 3.51 (ddd, J=13.4, 6.3, 4.6 Hz, 1H), 3.43–3.36 (m, 1H), 3.17 (d, J = 2.6 Hz, 2H); FTMS (ESI-TOF) *m/z*: $[M+H]^+$ Calcd for $C_{10}H_{10}Br_4N_3O_2^+$ m/z: 523.74601, Found 523.74584.

Synthesis of dual CK2/PIM-1 inhibitors - TBBi amino alcohol derivatives (4–11) – Summary

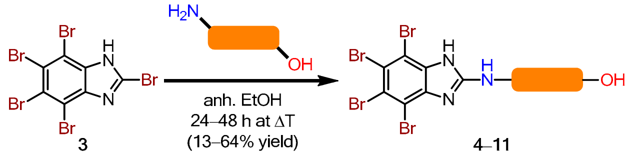

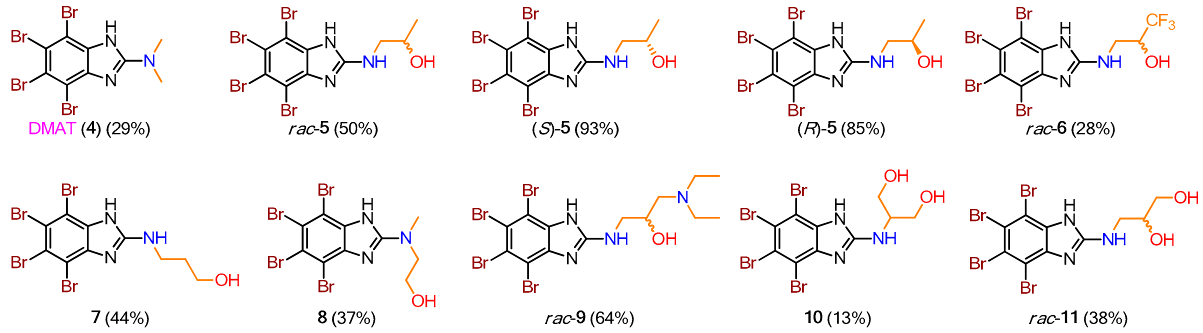

Table S1. Screening conditions for the synthesis of (2*S*)-1-[(4,5,6,7-tetrabromo-1*H*-benzimidazol-2-yl)amino]propan-2-ol [(*S*)-**5**] using commercially available (*S*)-1-aminopropan-2-ol (>99% ee).

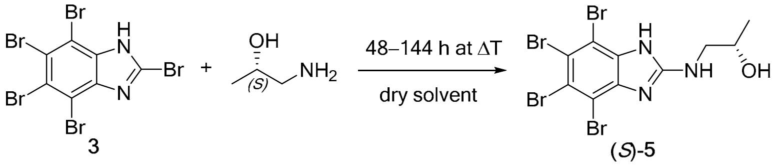

Entry	Product	T [°C]	*t* [h]	Yield[c] [%]	ee_p[d] [%]
1	1,4-Dioxane (–0.31)	60	144	N.D.[f]	26
2	DMF (–0.60)	153	72	N.D.[f]	83
3	EtOH (0.07)	78	72	N.D.[f]	39
4	CH_3CN (0.17)	82	72	N.D.[f]	81
5	$PhCH_3$ (2.52)	100	48	93	>99

[a] Reaction conditions: **3** (50 mg, 97.5 µmmol), (*S*)-1-aminopropan-2-ol (110 mg, 1.46 mmol, 113 µL, 15.0 equiv), solvent (1 mL), sealed-tube.
[b] Logarithm of the partition coefficient of a given solvent between *n*-octanol and water according to ChemBioDraw Ultra 13.0 software indications.
[c] Isolated yield after column chromatography eluted with a gradient of hexane/AcOEt (50:50, v/v) and then $CHCl_3$/MeOH (95:5, v/v) mixture.
[d] Determined by chiral HPLC analyses by using a Chiralcel AD-H column.
[f] Not determined due to low conversion.

3. Biological assays

3.1. Antibodies

The following primary antibodies were used: anti-GAPDH (Millipore, St. Louis, MO, USA; #MAB374, 1:1000, overnight, +4°C); anti-p-p65-Ser529 (Biorbyt Cambridge, UK; #orb14916, 1:500, overnight, +4°C), anti-NFkB p65 (Biorbyt Cambridge, UK; orb214507, 1:1000, overnight, +4°C); anti-BAD (Apoptosis I Sampler Kit, # 612741, BD Biosciences, San Diego, CA, USA; 1:1000, overnight, +4°C), anti-phospho-BAD-Ser112 (CST, Beverly, MA, USA; 9291S, 1:500 overnight, +4°C). The following secondary antibodies were used: HRP-conjugated anti-mouse (Dako, Santa Clara, CA, USA); #P0447, 1:1000, 1 h, RT; Milli-pore, St. Louis, MO, USA; #AP308P, 1:10000, 1 h, RT) and anti-rabbit IgG (Dako, Santa Clara, CA, USA); #P0448, 1:2000, 1h, RT; Millipore, St. Louis, MO, USA; #AP307P, 1:10000, 1 h, RT). Protease inhibitors were from Roche Applied Science (#11836153001, Mannheim, Germany). Nitrocellulose membrane was from GE Healthcare Life Sciences (Freiburg, Germany), and solvents for the HRP reaction (Western Bright Peroxide and Western Bright Quantum) were purchased from Advansta (Menlo Park, CA, USA).), ECL reagent was from BioRad (Hercules, CA, USA) and Millipore (St. Louis, MO, USA). Other solvents, reagents, and chemicals were purchased from POCH (Avantor Performance Materials, Gliwice, Poland), Merck and Sigma-Aldrich Chemical Company (St. Louis, MO, USA).

3.2. Cell Culture and Agents Treatment

Acute lymphoblastic leukemia ALL cell line(named CCRF-CEM) was purchased from the European Collection of Authenticated Cell Cultures (ECACC), whereas MCF-7 (hormone-dependent breast adenocarcinoma), K-562 (human chronic myelogenous leukemia) and Vero cells (*Cercopithecus aethiops* kidney) were purchased from the American Type Culture Collection (ATCC, Manassas, VA, USA). CCRF-CEM and K-562 cells were cultured in RPMI 1640, whereas MCF-7 cells were cultured in DMEM (Sigma-Aldrich Chemical Company, St. Louis, MO, USA). Vero cells were cultured in Minimum Essential Medium Eagle (Sigma-Aldrich Chemical Company, St. Louis, MO, USA). All the cell lines were supplemented with 10% fetal bovine serum (FBS, Sigma-Aldrich Chemical Company, St. Louis, MO, USA), 2 mM L-glutamine, and antibiotics (100 U/mL penicillin, 100 µg/mL streptomycin) (Sigma-Aldrich Chemical Company, St. Louis, MO, USA). Cells were grown in 75-cm^2 cell culture flasks (Sarstedt, Nümbrecht, Germany) in a humidified atmosphere of CO_2/air (5%/95%) at 37 °C. All the experiments were performed in exponentially growing cultures. Stock solutions of the test compounds were prepared in DMSO and stored at –80 °C for a maximum of one month. For the cytotoxicity studies, stock solutions of the tested compounds were diluted 400-fold with the proper culture medium to obtain the final concentrations. In turn, the final concentration of DMSO was not higher than 0.5%.

3.3. Western Blotting

The exponentially growing MCF-7 cells were seeded at 6 × 10^5 cells in 6 cm diameter plates. Subsequently, compounds were added in a final concentration of 0.5% DMSO. After incubation, floating cells were collected, and the cell monolayer was washed three times in ice-cold PBS. Next, the cells were scraped, combined with floating cells, and lysed in RIPA buffer consisting of 50 mM Tris-HCl (pH 7.4), 1% NP-40, 0.5% sodium deoxycholate, 0.1% SDS, 150 mM NaCl, 2 mM EDTA, 50 mM NaF, 0.2 mM sodium orthovanadate, and protease inhibitors cocktail. Subsequently, cells were centrifuged at 20,000 × g for 15 min at 4 °C, and the supernatants were collected and stored at –80 °C. Then, supernatants were incubated with the Laemmli buffer for 5 min at 98 °C. Equal amounts of total protein (20 µg) were analyzed

by SDS-PAGE, and subsequently, Western blotting was performed using pri-mary antibodies in blocking buffer containing either 3% BSA (bovine serum albumin;) or 3% milk in TBS-T (Tris-buffered saline (10 mM Tris-HCl (pH 8), 150 mM NaCl) containing 0.1% Tween 20 or 0.2% Triton X-100) for 1 h. After overnight incubation at 4 °C with the primary antibodies, the membranes were washed with TBS-T and probed with respect to secondary antibodies. The ECL substrate was used for detection, and immunoblots were scanned using G Box Chemi (Syngene, Cambridge, UK) with GeneSys software (Syngene, Cambridge, UK).

3.4. Inhibition of CK2 and PIM-1 kinases by the tested compounds

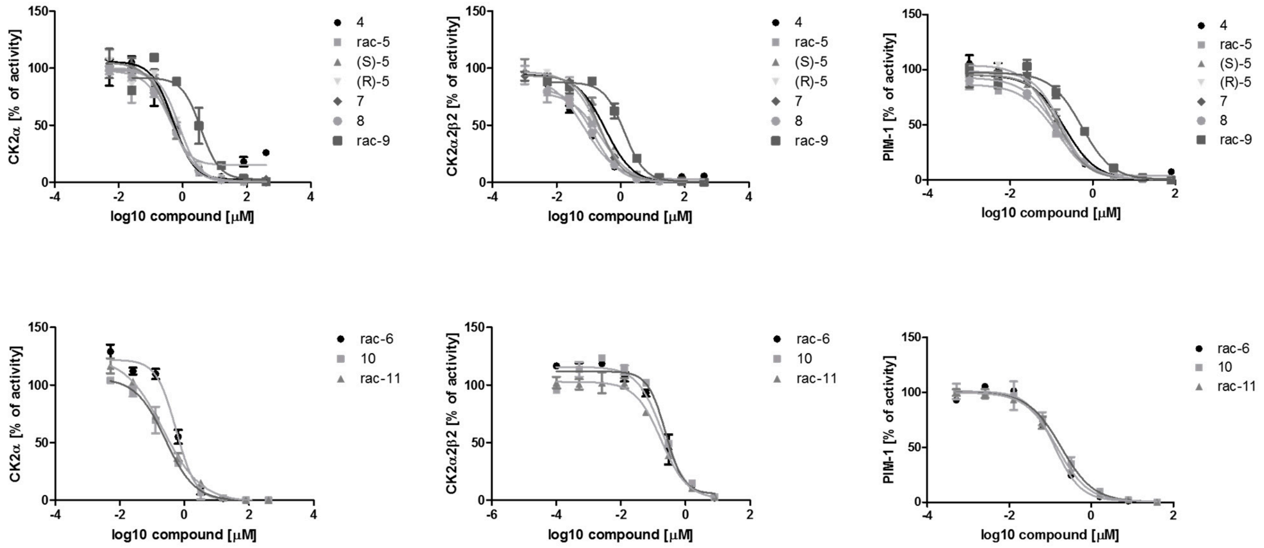

Figure S2. Sigmoidal dose-response curves. Inhibition of human CK2 catalytic subunit (CK2α), CK2 holoenzyme (CK2 α2β2), and PIM-1 kinases by the newly synthesized compounds were evaluated using the radiometric assay. The synthetic peptide RRRADDSDDDDD was used as the substrate of CK2, and peptide ARKRRRHPSGPPTA as the substrate of PIM-1. The experimental data were fitted to sigmoidal dose-response (variable slope) Y = Bottom + (Top-Bottom) / (1+10^((LogIC50-X)*HillSlope) equation in GraphPad Prism.

3.5. Induction of apoptosis in CCRF-CEM, K-562, and MCF-7 cells

Table S2. The data were determined by FACSCanto II flow cytometer after 48 h of treatment with compounds **4** and *rac*-**6**. Cells were stained with annexin V-FITC and PI (propidium iodide); Mean and standard deviation (SD) of necrosis, viable and early and late apoptosis as percent from three independent experiments each.

Cell line	Compound	The concentration of compound [µM]	Necrosis [%]	Late apoptosis [%]	Viable [%]	Early apoptosis [%]
MCF-7	Control	0	13.00±5.21	11.86±4.72	74.28±8.76	0.98±0.48
	**4**	5	11.62±2.01	8.44±1.47	78.96±2.76	0.98±0.62
		10	11.96±2.46	15.94±3.58	70.32±3.36	1.80±1.31
	rac-**6**	5	8.94±1.14	10.96±1.63	78.96±1.94	1.18±1.08
		10	8.28±1.69	19.05±7.40	69.08±13.07	3.53±5.45
CCRF-CEM	Control	0	0.95±0.92	11.90±0.28	85.10±0.85	2.05±0.35
	**4**	5	1.45±0.07	13.55±0.35	83.30±0.57	1.65±0.78
		10	1.35±0.49	16.75±0.21	80.35±0.78	1.55±0.07
	rac-**6**	5	2.45±0.07	18.85±3.61	75.95±3.75	2.75±0.07
		10	5.00±1.41	44.05±4.03	48.60±2.26	2.30±0.42
K-562	Control	0	1.10±0.44	6.87±0.81	90.43±0.93	1.67±0.31
	**4**	5	1.05±0.69	4.70±1.21	92.33±0.99	1.95±0.61
		10	0.67±0.31	7.43±3.52	90.60±4.27	1.33±0.67
	rac-**6**	5	0.63±0.06	5.97±1.02	87.97±0.81	5.13±0.40
		10	1.70±0.60	10.80±4.06	82.83±3.68	4.67±1.21

4. Evaluation of ADMET profile for the obtained compounds.

Absorption, distribution, metabolism, excretion, and toxicity (ADMET) profiles for the synthesized compounds were evaluated using the SwissADME web platform (http://www.swissadme.ch/) with a web-based interface provided by the Molecular Modeling Group of the Swiss Institute of Bioinformatics (SIB) [4].

Molecule 1

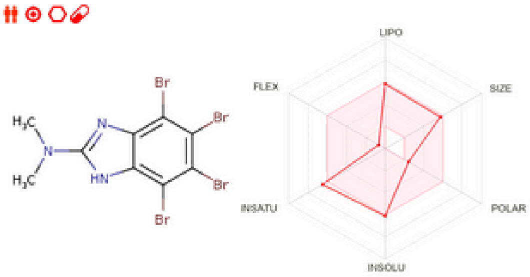

SMILES CN(c1[nH]c2c(n1)c(Br)c(c(c2Br)Br)Br)C

Physicochemical Properties	
Formula	C9H7Br4N3
Molecular weight	476.79 g/mol
Num. heavy atoms	16
Num. arom. heavy atoms	9
Fraction Csp3	0.22
Num. rotatable bonds	1
Num. H-bond acceptors	1
Num. H-bond donors	1
Molar Refractivity	81.10
TPSA	31.92 Å²

Lipophilicity	
Log $P_{o/w}$ (iLOGP)	2.79
Log $P_{o/w}$ (XLOGP3)	4.71
Log $P_{o/w}$ (WLOGP)	4.68
Log $P_{o/w}$ (MLOGP)	4.17
Log $P_{o/w}$ (SILICOS-IT)	4.25
Consensus Log $P_{o/w}$	4.12

Water Solubility	
Log *S* (ESOL)	-6.11
Solubility	3.67e-04 mg/ml ; 7.70e-07 mol/l
Class	Poorly soluble
Log *S* (Ali)	-5.11
Solubility	3.71e-03 mg/ml ; 7.78e-06 mol/l
Class	Moderately soluble
Log *S* (SILICOS-IT)	-6.28
Solubility	2.52e-04 mg/ml ; 5.28e-07 mol/l
Class	Poorly soluble

Pharmacokinetics	
GI absorption	High
BBB permeant	Yes
P-gp substrate	No
CYP1A2 inhibitor	No
CYP2C19 inhibitor	No
CYP2C9 inhibitor	Yes
CYP2D6 inhibitor	No
CYP3A4 inhibitor	No
Log K_p (skin permeation)	-5.86 cm/s

Druglikeness	
Lipinski	Yes; 1 violation: MLOGP>4.15
Ghose	Yes
Veber	Yes
Egan	Yes
Muegge	Yes
Bioavailability Score	0.55

Medicinal Chemistry	
PAINS	0 alert
Brenk	2 alerts: halogenated_ring_1, halogenated_ring_2
Leadlikeness	No; 2 violations: MW>350, XLOGP3>3.5
Synthetic accessibility	2.27

Molecule 2

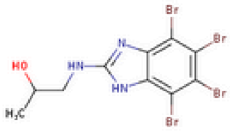

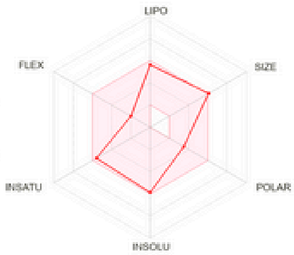

SMILES CC(CNc1[nH]c2c(n1)c(Br)c(c(c2Br)Br)Br)O

Physicochemical Properties	
Formula	C10H9Br4N3O
Molecular weight	506.81 g/mol
Num. heavy atoms	18
Num. arom. heavy atoms	9
Fraction Csp3	0.30
Num. rotatable bonds	3
Num. H-bond acceptors	2
Num. H-bond donors	3
Molar Refractivity	86.98
TPSA	60.94 Å²

Lipophilicity	
Log $P_{o/w}$ (iLOGP)	2.55
Log $P_{o/w}$ (XLOGP3)	4.32
Log $P_{o/w}$ (WLOGP)	4.21
Log $P_{o/w}$ (MLOGP)	3.57
Log $P_{o/w}$ (SILICOS-IT)	4.28
Consensus Log $P_{o/w}$	3.79

Water Solubility	
Log S (ESOL)	-5.88
Solubility	6.75e-04 mg/ml ; 1.33e-06 mol/l
Class	Moderately soluble
Log S (Ali)	-5.31
Solubility	2.46e-03 mg/ml ; 4.86e-06 mol/l
Class	Moderately soluble
Log S (SILICOS-IT)	-6.45
Solubility	1.79e-04 mg/ml ; 3.54e-07 mol/l
Class	Poorly soluble

Pharmacokinetics	
GI absorption	High
BBB permeant	Yes
P-gp substrate	No
CYP1A2 inhibitor	No
CYP2C19 inhibitor	No
CYP2C9 inhibitor	Yes
CYP2D6 inhibitor	Yes
CYP3A4 inhibitor	Yes
Log K_p (skin permeation)	-6.32 cm/s

Druglikeness	
Lipinski	Yes; 1 violation: MW>500
Ghose	No; 1 violation: MW>480
Veber	Yes
Egan	Yes
Muegge	Yes
Bioavailability Score	0.55

Medicinal Chemistry	
PAINS	0 alert
Brenk	2 alerts: halogenated_ring_1, halogenated_ring_2
Leadlikeness	No; 2 violations: MW>350, XLOGP3>3.5
Synthetic accessibility	2.77

Molecule 3

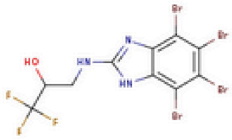

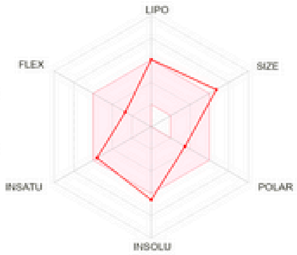

SMILES Brc1c(Br)c(Br)c(c2c1nc([nH]2)NCC(C(F)(F)F)O)Br

Physicochemical Properties	
Formula	C10H6Br4F3N3O
Molecular weight	560.79 g/mol
Num. heavy atoms	21
Num. arom. heavy atoms	9
Fraction Csp3	0.30
Num. rotatable bonds	4
Num. H-bond acceptors	5
Num. H-bond donors	3
Molar Refractivity	87.17
TPSA	60.94 Å²

Lipophilicity	
Log $P_{o/w}$ (iLOGP)	2.10
Log $P_{o/w}$ (XLOGP3)	5.06
Log $P_{o/w}$ (WLOGP)	6.02
Log $P_{o/w}$ (MLOGP)	3.93
Log $P_{o/w}$ (SILICOS-IT)	4.92
Consensus Log $P_{o/w}$	4.41

Water Solubility	
Log S (ESOL)	-6.56
Solubility	1.55e-04 mg/ml ; 2.77e-07 mol/l
Class	Poorly soluble
Log S (Ali)	-6.08
Solubility	4.65e-04 mg/ml ; 8.29e-07 mol/l
Class	Poorly soluble
Log S (SILICOS-IT)	-6.88
Solubility	7.31e-05 mg/ml ; 1.30e-07 mol/l
Class	Poorly soluble

Pharmacokinetics	
GI absorption	High
BBB permeant	No
P-gp substrate	No
CYP1A2 inhibitor	No
CYP2C19 inhibitor	No
CYP2C9 inhibitor	Yes
CYP2D6 inhibitor	No
CYP3A4 inhibitor	Yes
Log K_p (skin permeation)	-6.13 cm/s

Druglikeness	
Lipinski	Yes; 1 violation: MW>500
Ghose	No; 2 violations: MW>480, WLOGP>5.6
Veber	Yes
Egan	No; 1 violation: WLOGP>5.88
Muegge	No; 1 violation: XLOGP3>5
Bioavailability Score	0.55

Medicinal Chemistry	
PAINS	0 alert
Brenk	2 alerts: halogenated_ring_1, halogenated_ring_2
Leadlikeness	No; 2 violations: MW>350, XLOGP3>3.5
Synthetic accessibility	2.78

Molecule 4

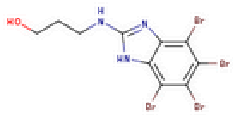

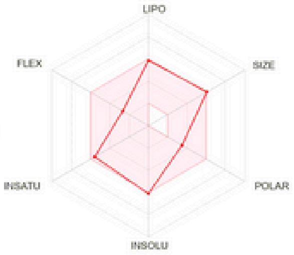

SMILES OCCCNc1[nH]c2c(n1)c(Br)c(c(c2Br)Br)Br

Physicochemical Properties	
Formula	C10H9Br4N3O
Molecular weight	506.81 g/mol
Num. heavy atoms	18
Num. arom. heavy atoms	9
Fraction Csp3	0.30
Num. rotatable bonds	4
Num. H-bond acceptors	2
Num. H-bond donors	3
Molar Refractivity	86.98
TPSA	60.94 Å²

Lipophilicity	
Log $P_{o/w}$ (iLOGP)	2.32
Log $P_{o/w}$ (XLOGP3)	4.79
Log $P_{o/w}$ (WLOGP)	4.22
Log $P_{o/w}$ (MLOGP)	3.57
Log $P_{o/w}$ (SILICOS-IT)	4.45
Consensus Log $P_{o/w}$	3.87

Water Solubility	
Log *S* (ESOL)	-6.11
Solubility	3.97e-04 mg/ml ; 7.84e-07 mol/l
Class	Poorly soluble
Log *S* (Ali)	-5.80
Solubility	8.00e-04 mg/ml ; 1.58e-06 mol/l
Class	Moderately soluble
Log *S* (SILICOS-IT)	-6.82
Solubility	7.60e-05 mg/ml ; 1.50e-07 mol/l
Class	Poorly soluble

Pharmacokinetics	
GI absorption	High
BBB permeant	Yes
P-gp substrate	No
CYP1A2 inhibitor	Yes
CYP2C19 inhibitor	No
CYP2C9 inhibitor	Yes
CYP2D6 inhibitor	Yes
CYP3A4 inhibitor	Yes
Log K_p (skin permeation)	-5.99 cm/s

Druglikeness	
Lipinski	Yes; 1 violation: MW>500
Ghose	No; 1 violation: MW>480
Veber	Yes
Egan	Yes
Muegge	Yes
Bioavailability Score	0.55

Medicinal Chemistry	
PAINS	0 alert
Brenk	2 alerts: halogenated_ring_1, halogenated_ring_2
Leadlikeness	No; 2 violations: MW>350, XLOGP3>3.5
Synthetic accessibility	2.32

Molecule 5

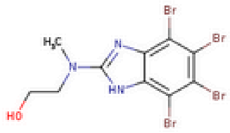

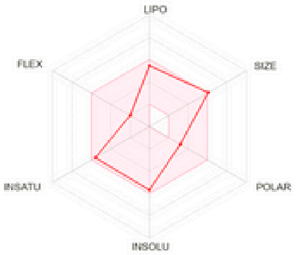

SMILES OCCN(c1[nH]c2c(n1)c(Br)c(c(c2Br)Br)Br)C

Physicochemical Properties	
Formula	C10H9Br4N3O
Molecular weight	506.81 g/mol
Num. heavy atoms	18
Num. arom. heavy atoms	9
Fraction Csp3	0.30
Num. rotatable bonds	3
Num. H-bond acceptors	2
Num. H-bond donors	2
Molar Refractivity	87.07
TPSA	52.15 Å²

Lipophilicity	
Log $P_{o/w}$ (iLOGP)	2.69
Log $P_{o/w}$ (XLOGP3)	4.02
Log $P_{o/w}$ (WLOGP)	4.04
Log $P_{o/w}$ (MLOGP)	3.57
Log $P_{o/w}$ (SILICOS-IT)	4.01
Consensus Log $P_{o/w}$	3.67

Water Solubility	
Log *S* (ESOL)	-5.69
Solubility	1.04e-03 mg/ml ; 2.06e-06 mol/l
Class	Moderately soluble
Log *S* (Ali)	-4.82
Solubility	7.71e-03 mg/ml ; 1.52e-05 mol/l
Class	Moderately soluble
Log *S* (SILICOS-IT)	-6.09
Solubility	4.10e-04 mg/ml ; 8.09e-07 mol/l
Class	Poorly soluble

Pharmacokinetics	
GI absorption	High
BBB permeant	Yes
P-gp substrate	No
CYP1A2 inhibitor	Yes
CYP2C19 inhibitor	No
CYP2C9 inhibitor	Yes
CYP2D6 inhibitor	Yes
CYP3A4 inhibitor	No
Log K_p (skin permeation)	-6.54 cm/s

Druglikeness	
Lipinski	Yes; 1 violation: MW>500
Ghose	No; 1 violation: MW>480
Veber	Yes
Egan	Yes
Muegge	Yes
Bioavailability Score	0.55

Medicinal Chemistry	
PAINS	0 alert
Brenk	2 alerts: halogenated_ring_1, halogenated_ring_2
Leadlikeness	No; 2 violations: MW>350, XLOGP3>3.5
Synthetic accessibility	2.37

Molecule 6

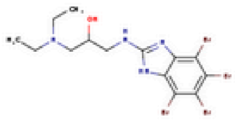

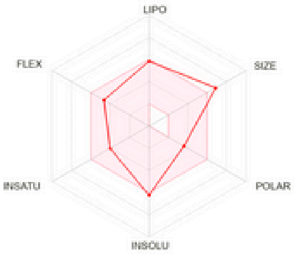

SMILES CCN(CC(CNc1[nH]c2c(n1)c(Br)c(c(c2Br)Br)Br)O)CC

Physicochemical Properties	
Formula	C14H18Br4N4O
Molecular weight	577.93 g/mol
Num. heavy atoms	23
Num. arom. heavy atoms	9
Fraction Csp3	0.50
Num. rotatable bonds	7
Num. H-bond acceptors	3
Num. H-bond donors	3
Molar Refractivity	109.10
TPSA	64.18 Å²

Lipophilicity	
Log $P_{o/w}$ (iLOGP)	3.68
Log $P_{o/w}$ (XLOGP3)	4.72
Log $P_{o/w}$ (WLOGP)	4.54
Log $P_{o/w}$ (MLOGP)	3.68
Log $P_{o/w}$ (SILICOS-IT)	4.58
Consensus Log $P_{o/w}$	4.24

Water Solubility	
Log *S* (ESOL)	-6.22
Solubility	3.45e-04 mg/ml ; 5.97e-07 mol/l
Class	Poorly soluble
Log *S* (Ali)	-5.80
Solubility	9.22e-04 mg/ml ; 1.60e-06 mol/l
Class	Moderately soluble
Log *S* (SILICOS-IT)	-7.30
Solubility	2.89e-05 mg/ml ; 5.00e-08 mol/l
Class	Poorly soluble

Pharmacokinetics	
GI absorption	High
BBB permeant	Yes
P-gp substrate	No
CYP1A2 inhibitor	Yes
CYP2C19 inhibitor	No
CYP2C9 inhibitor	No
CYP2D6 inhibitor	Yes
CYP3A4 inhibitor	Yes
Log K_p (skin permeation)	-6.47 cm/s

Druglikeness	
Lipinski	Yes; 1 violation: MW>500
Ghose	No; 1 violation: MW>480
Veber	Yes
Egan	Yes
Muegge	Yes
Bioavailability Score	0.55

Medicinal Chemistry	
PAINS	0 alert
Brenk	2 alerts: halogenated_ring_1, halogenated_ring_2
Leadlikeness	No; 2 violations: MW>350, XLOGP3>3.5
Synthetic accessibility	3.26

Molecule 7

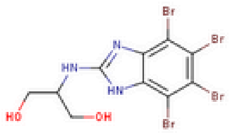

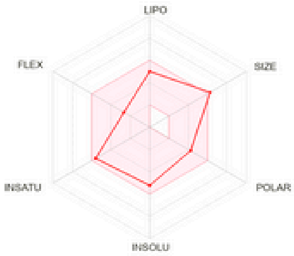

SMILES OCC(Nc1[nH]c2c(n1)c(Br)c(c(c2Br)Br)Br)CO

Physicochemical Properties	
Formula	C10H9Br4N3O2
Molecular weight	522.81 g/mol
Num. heavy atoms	19
Num. arom. heavy atoms	9
Fraction Csp3	0.30
Num. rotatable bonds	4
Num. H-bond acceptors	3
Num. H-bond donors	4
Molar Refractivity	88.14
TPSA	81.17 Å²

Lipophilicity	
Log $P_{o/w}$ (iLOGP)	2.27
Log $P_{o/w}$ (XLOGP3)	3.27
Log $P_{o/w}$ (WLOGP)	3.19
Log $P_{o/w}$ (MLOGP)	2.74
Log $P_{o/w}$ (SILICOS-IT)	3.70
Consensus Log $P_{o/w}$	3.03

Water Solubility	
Log *S* (ESOL)	-5.23
Solubility	3.09e-03 mg/ml ; 5.91e-06 mol/l
Class	Moderately soluble
Log *S* (Ali)	-4.65
Solubility	1.17e-02 mg/ml ; 2.24e-05 mol/l
Class	Moderately soluble
Log *S* (SILICOS-IT)	-5.88
Solubility	6.97e-04 mg/ml ; 1.33e-06 mol/l
Class	Moderately soluble

Pharmacokinetics	
GI absorption	High
BBB permeant	No
P-gp substrate	No
CYP1A2 inhibitor	Yes
CYP2C19 inhibitor	No
CYP2C9 inhibitor	No
CYP2D6 inhibitor	Yes
CYP3A4 inhibitor	Yes
Log K_p (skin permeation)	-7.17 cm/s

Druglikeness	
Lipinski	Yes; 1 violation: MW>500
Ghose	No; 1 violation: MW>480
Veber	Yes
Egan	Yes
Muegge	Yes
Bioavailability Score	0.55

Medicinal Chemistry	
PAINS	0 alert
Brenk	2 alerts: halogenated_ring_1, halogenated_ring_2
Leadlikeness	No; 1 violation: MW>350
Synthetic accessibility	2.41

Molecule 8

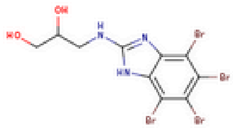

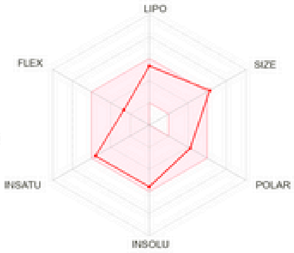

SMILES OCC(CNc1[nH]c2c(n1)c(Br)c(c(c2Br)Br)Br)O

Physicochemical Properties	
Formula	C10H9Br4N3O2
Molecular weight	522.81 g/mol
Num. heavy atoms	19
Num. arom. heavy atoms	9
Fraction Csp3	0.30
Num. rotatable bonds	4
Num. H-bond acceptors	3
Num. H-bond donors	4
Molar Refractivity	88.14
TPSA	81.17 Å²

Lipophilicity	
Log $P_{o/w}$ (iLOGP)	1.85
Log $P_{o/w}$ (XLOGP3)	3.82
Log $P_{o/w}$ (WLOGP)	3.19
Log $P_{o/w}$ (MLOGP)	2.74
Log $P_{o/w}$ (SILICOS-IT)	3.70
Consensus Log $P_{o/w}$	3.06

Water Solubility	
Log *S* (ESOL)	-5.57
Solubility	1.39e-03 mg/ml ; 2.66e-06 mol/l
Class	Moderately soluble
Log *S* (Ali)	-5.22
Solubility	3.15e-03 mg/ml ; 6.03e-06 mol/l
Class	Moderately soluble
Log *S* (SILICOS-IT)	-5.88
Solubility	6.97e-04 mg/ml ; 1.33e-06 mol/l
Class	Moderately soluble

Pharmacokinetics	
GI absorption	High
BBB permeant	No
P-gp substrate	No
CYP1A2 inhibitor	Yes
CYP2C19 inhibitor	No
CYP2C9 inhibitor	No
CYP2D6 inhibitor	Yes
CYP3A4 inhibitor	Yes
Log K_p (skin permeation)	-6.78 cm/s

Druglikeness	
Lipinski	Yes; 1 violation: MW>500
Ghose	No; 1 violation: MW>480
Veber	Yes
Egan	Yes
Muegge	Yes
Bioavailability Score	0.55

Medicinal Chemistry	
PAINS	0 alert
Brenk	2 alerts: halogenated_ring_1, halogenated_ring_2
Leadlikeness	No; 2 violations: MW>350, XLOGP3>3.5
Synthetic accessibility	2.86

5. Molecular docking.

5.1. Molecular docking preparation

Molecular docking studies to establish favorable ligand binding geometries for both studied inhibitors, namely 1,1,1-trifluoro-3-[(4,5,6,7-tetrabromo-1*H*-benzimidazol-2-yl)amino]propan-2-ol (*rac*-**6**) and 3-[(4,5,6,7-tetrabromo-1*H*-benzimidazol-2-yl)amino]propane-1,2-diol (*rac*-**11**), were carried out on a 24 CPUs-based desktop PC computer equipped with AMD Ryzen™ 9 3900X 12-Core Processor 3800 MHz and 32 GB of RAM on a Microsoft Windows 11 Professional 64-bit operating system using AutoDock Vina vs. 1.1.2 program for Windows (http://autodock.scripps.edu/) [4]. At first, the respective ligand molecules *rac*-**6** and *rac*-**11** in non-ionizable form were prepared with ChemAxon MarvinSketch vs. 14.9.1.0 (http://www.chemaxon.com/marvin/) using general 'Cleaning in 3D' option to assign with proper 3D orientation and then calculating conformers with MMFF94 force field parameters and saved as .pdb file. (*Attention:* we have prepared ligands in non-ionizable form since the non-protonated forms of TBBi-derivatives are expected to be largely predominant in solution at physiological pH). To obtain the minimum energy conformation for docking studies, the initial geometries of the afore-pretreated ligands were additionally optimized in Avogadro vs. 1.2.0. (http://avogadro.cc/) using MMFF94 force field with 500 steps and Steepest Descent Algorithm, after adding all the hydrogens to the structure, and saved as .mol2 files. The energy of the ligand molecules was minimized using the built-in feature of Avogadro, including the General Amber Force Field (GAFF) [5] with the Steepest Descent Algorithm (100 steps). The minimum conformation energies obtained for each ligand were as follows: $E_{calc.}$ = –226.758 kJ/mol for *rac*-**6** and $E_{calc.}$ = –171.375 kJ/mol for *rac*-**11**. The visualization of the optimized geometries was performed using the molecular visualization software POV-Ray for Windows vs. 3.7.0.msvc10.win64 licensed under the terms of the GNU Affero General Public License (AGPL3) (**Figure 2**). Afterward, the Gasteiger partial charges were calculated with AutoDock Tools vs. 1.5.6 (ADT, S3 http://mgltools.scripps.edu/). In contrast, all torsion angles for each ligand were considered flexible, and all the possible rotatable bonds and non-polar hydrogens were determined. The final ligands' files were saved as PDBQT files (.pdbqt format) and were ready for the docking procedure disclosed in section *4.2. Molecular docking procedure*.

The crystal structures of human protein kinases, namely CK2-α (PDB code: 4KWP) [6] of the highest available resolution (1.25 Å) and PIM-1 (PDB code: 4DTK) [7] with the resolution (1.86 Å), were downloaded from Brookhaven RCSB Protein Data Bank (PDB

database, http://www.rcsb.org/pdb/). To avoid steric clashes within the model, the crude target proteins .pdb files were prepared by UCSF Chimera vs. 1.11.2 package (http://www.cgl.ucsf.edu/chimera/) [8] by removing all nonstandard molecules, including 4,5,6,7-tetrabromo-1-(2-deoxy-beta-*D*-erythro-pentofuranosyl)-1*H*-benzimidazole (EXX), sulfate ion (SO_4), 1,2-ethanediol (EDO), triethylene glycol (PGE), di(hydroxyethyl)ether (PEG), and dimethyl sulfoxide (DMS) in the case of 4KWP as well as (5*Z*)-5-{2-[(3*R*)-3-aminopiperidin-1-yl]-3-(propan-2-yloxy)benzylidene}-1,3-thiazolidine-2,4-dione (7LI), EDO, and SO_4 in the case of 4DTK, respectively. Next, the polar hydrogen atoms were added, and Gasteiger charges were calculated with AutoDock Tools 1.5.6 package using its standard utility scripts, and then the final protein files were saved as PDBQT files (.pdbqt). Next, a searching 'grid box' was set by using the AutoGrid function to perform docking in a (40 × 40 × 40 Å)-unit grid box (final size space dimension: x = 40 Å, y = 40 Å, z = 40 Å), centered on catalytic cavity located in CK2-α (center_x = 21.580; center_y = −31.180; center_z = 13.278) or ATP binding site of the PIM-1 (center_x = 18.633; center_y = −35.091; center_z = −1.101) as target coordinates with a grid spacing of 0.325 Å, respectively.

5.2. Molecular docking procedure

Docking was performed into a rigid protein as well as using advanced protein flexibility by specifying flexible sidechains. Each docking was performed with an exhaustiveness level of 96 concerning global search. For each ligand molecule, 100 independent runs were performed using the Lamarckian Genetic Algorithm (GA) with at most 106 energy evaluations and a maximum number of generations of >27 000 Å^3 (the search space volume). The rest of the docking parameters, including the remaining Lamarckian GA parameters, were set as default using the standard values for genetic Vina algorithms (the posed dockings were below 5.00 Å rmsd). For validation of the docking protocol and the subsequent analysis of the docking results, two prominent kinase inhibitors which were crystalized with 4KWP and 4DTK, i.e., 4,5,6,7-tetrabromo-1-(2-deoxy-beta-*D*-erythro-pentofuranosyl)-1*H*-benzimidazole (EXX) for CK2-α and (5*Z*)-5-{2-[(3*R*)-3-aminopiperidin-1-yl]-3-(propan-2-yloxy)benzylidene}-1,3-thiazolidine-2,4-dione (7LI) for PIM-1, were docked as control ligands. The docking modes of each studied ligand [i.e., 1,1,1-trifluoro-3-[(4,5,6,7-tetrabromo-1*H*-benzimidazol-2-yl)amino]propan-2-ol (*rac*-**6**) and 3-[(4,5,6,7-tetrabromo-1*H*-benzimidazol-2-yl)amino]propane-1,2-diol (*rac*-**11**)] were clustered and ranked based on a mutual ligand–protein affinity expressed as absolute free binding energies [ΔG_{calc} (kcal/mol)] as well as the

values of root mean square deviation (rmsd) in both modes regarding rmsd lower bound (l.b.), and rmsd upper bound (u.b.), respectively. The rmsd-values were computed referring to the input structure submitted to docking simulations. For CK2-α (PDB code: 4KWP), the used random seed amounted to +1342461868 for *rac*-**6** and –2037069392 for *rac*-**11**; whereas for PIM-1 (PDB code: 4DTK), the used random seed amounted to +956047904 for *rac*-**6** and –769683352 for *rac*-**11**, respectively. The best nine poses (modes) were selected according to AutoDock Vina scoring functions mainly based on binding energies and showed mutual ligand–protein affinity (kcal/mol). The results of docking scoring of the respective ligands to protein kinases, CK2-α (PDB code: 4KWP) and PIM-1 (PDB code: 4DTK), are collected in **Table S2**. The results generated by AutoDock Vina, including optimized binding poses of all ligands in hypothetical complexes with CK2-α and PIM-1 proteins as well as critical polar contacts between the respective atoms of those ligands and receptor molecules (4KWP and 4DTK), were visualized using The PyMOL Molecular Graphics System software, vs. 1.3, Schrödinger, LLC (https://www.pymol.org/) (**Figure 9**).

Table S3. Docking scoring of the respective ligands (*rac*-**6** and *rac*-**11**) complexed with human protein kinases.

**Entry**	**Ligand**	**Protein kinase**	**Pose**[a]	**Affinity (kcal/mol)**	**Distance from best mode**[b]	
					**rmsd l.b.**	**rmsd u.b.**
1		CK2-α (PDB ID: 4KWP)	S1	–7.8	0.000	0.000
2			S2	–7.6	1.581	1.822
3			S3	–7.3	0.138	2.760
4			S4	–7.0	4.298	6.115
5			S5	–6.8	1.713	3.404
6			S6	–6.7	4.690	6.854
7			S7	–6.4	5.666	8.214
8			S8	–6.4	4.473	6.695
9	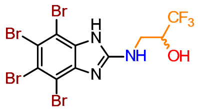		S9	–6.1	4.714	7.200
10	*rac*-6	PIM-1 (PDB ID: 4DTK)	S1	–8.4	0.000	0.000
11			S2	–8.4	0.218	2.764
12			S3	–7.2	1.719	2.503
13			S4	–7.2	6.371	8.228
14			S5	–7.1	6.314	8.475
15			S6	–7.1	6.163	8.352
16			S7	–7.0	6.796	8.635
17			S8	–7.0	5.893	7.679
18			S9	–6.8	4.705	6.571
19		CK2-α (PDB ID: 4KWP)	S1	–7.1	0.000	0.000
20			S2	–6.7	0.168	2.765
21			S3	–5.9	3.116	3.986
22			S4	–5.8	5.920	8.502
23			S5	–5.6	2.938	4.419
24			S6	–5.5	5.698	7.974
25			S7	–5.5	3.274	4.812
26			S8	–5.3	6.296	7.412
27	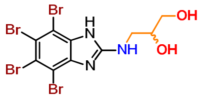		S9	–5.3	19.563	20.333
28	*rac*-11	PIM-1 (PDB ID: 4DTK)	S1	–7.4	0.000	0.000
29			S2	–7.3	0.090	2.762
30			S3	–7.2	1.499	1.907
31			S4	–7.1	5.440	8.159
32			S5	–7.0	5.113	7.426
33			S6	–6.9	5.105	7.819
34			S7	–6.8	4.849	7.025
35			S8	–6.6	4.063	6.286
36			S9	–6.4	4.372	7.353

[a] The pose S1 represents the lowest value of ΔG_{calc} (kcal/mol), which means that ligand-binding affinity to receptor is the highest, and in contrary, the S9 mode represent the lowest ligand-binding affinity.

[b] The values <2.000 rmsd represent the closest distance between the ligand and the opioid receptor binding site.

6. Analytical data (copies of HPLC chromatograms).

HPLC of *rac*-5 on Chiralpak AD-H at 25 °C

Conditions: *n*-hexane-2-PrOH (95:5, v/v); f=0.8 mL/min; λ=225 nm; *T*=25 °C

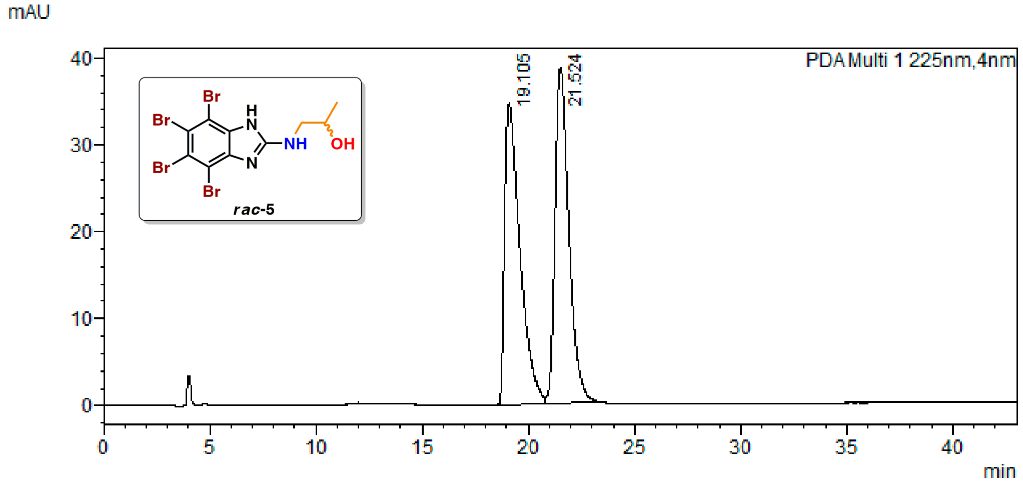

Peak Table

PDA Ch1 225nm

Peak#	Ret. Time	Area	Height	Height%	Area%
1	19.105	1743494	34652	47.356	49.883
2	21.524	1751653	38521	52.644	50.117
Total		3495148	73173	100.000	100.000

HPLC of (*S*)-5 (>99% ee) on Chiralpak AD-H at 25 °C

Conditions: *n*-hexane-2-PrOH (95:5, v/v); f=0.8 mL/min; λ=225 nm; *T*=25 °C

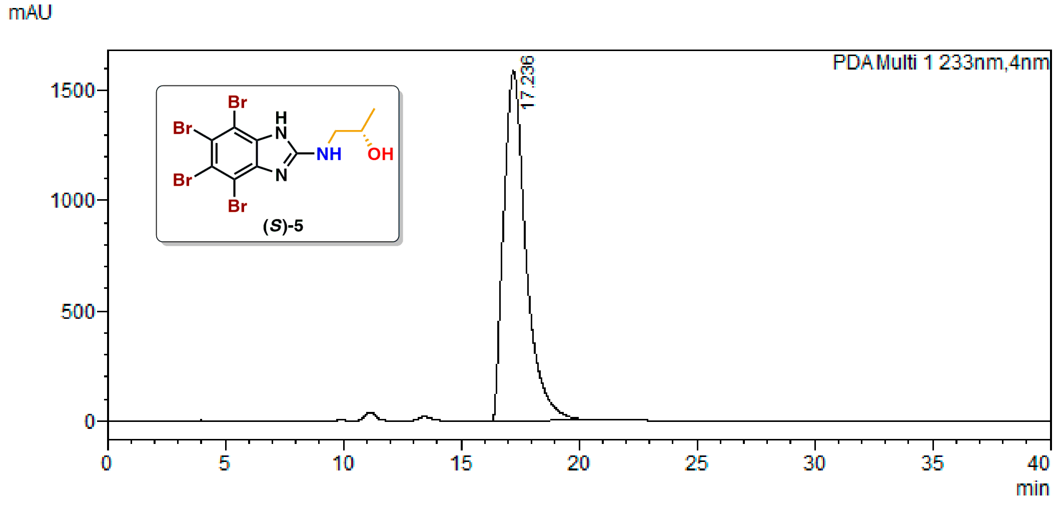

Peak Table

PDA Ch1 233nm

Peak#	Ret. Time	Area	Height	Height%	Area%
1	17.236	104461077	1589807	100.000	100.000
Total		104461077	1589807	100.000	100.000

HPLC of (*R*)-5 (>99% ee) on Chiralpak AD-H at 25 °C

Conditions: *n*-hexane-2-PrOH (95:5, v/v); f=0.8 mL/min; λ=225 nm; *T*=25 °C

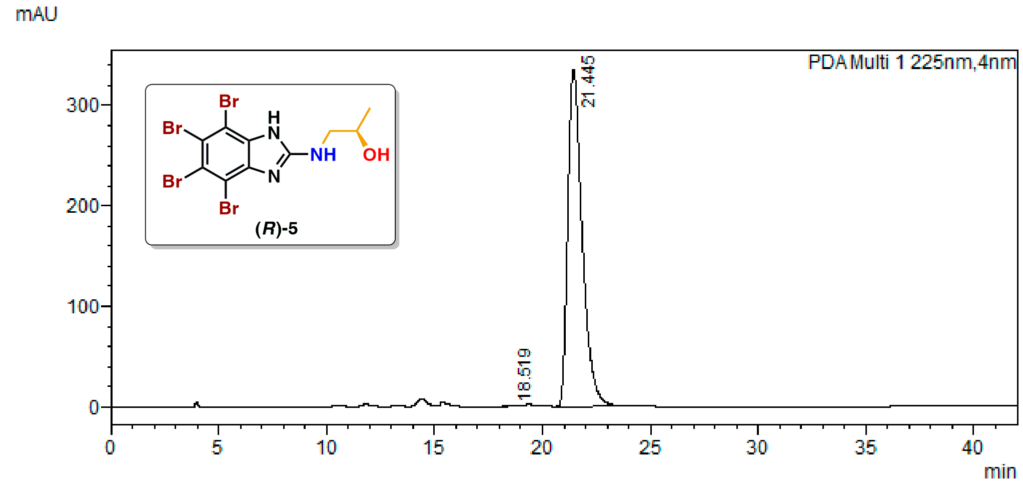

Peak Table

PDA Ch1 225nm

Peak#	Ret. Time	Area	Height	Height%	Area%
1	18.519	19195	693	0.206	0.123
2	21.445	15583513	334882	99.794	99.877
Total		15602709	335575	100.000	100.000

7. Spectral data (copies of NMR and FTMS spectra).

2-Bromo-1H-benzimidazole (2)

^{1}H NMR spectrum of **2** (500 MHz, DMSO-d_6)

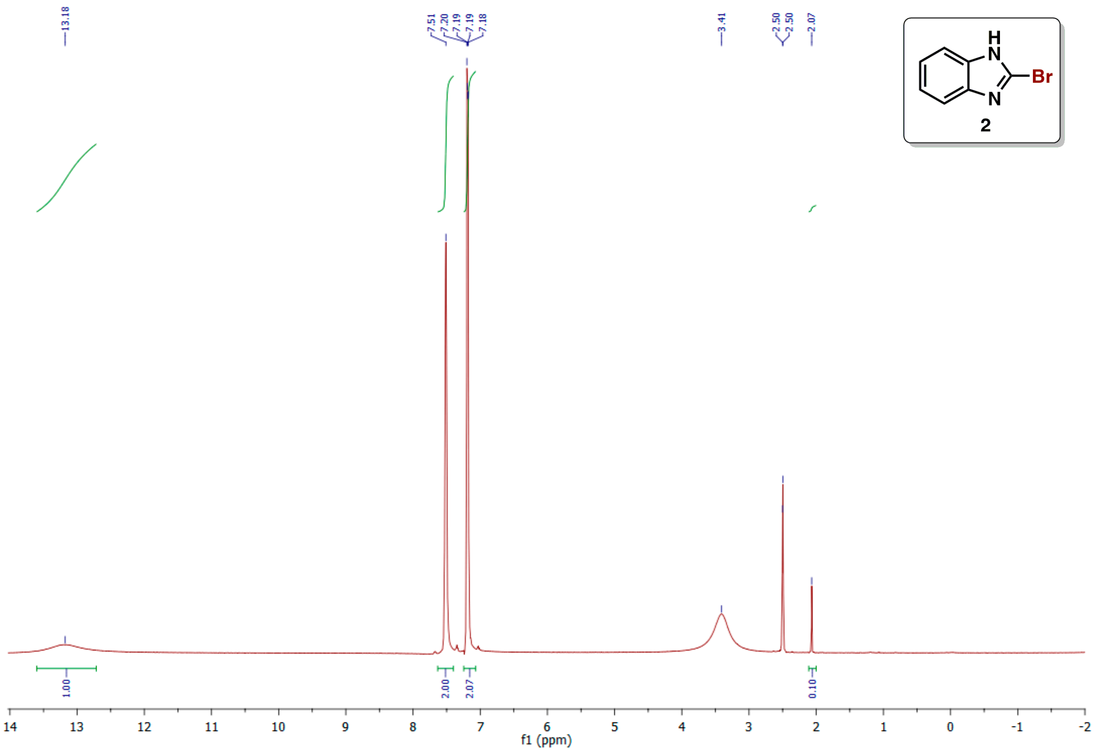

^{13}C NMR spectrum of **2** (126 MHz, DMSO-d_6)

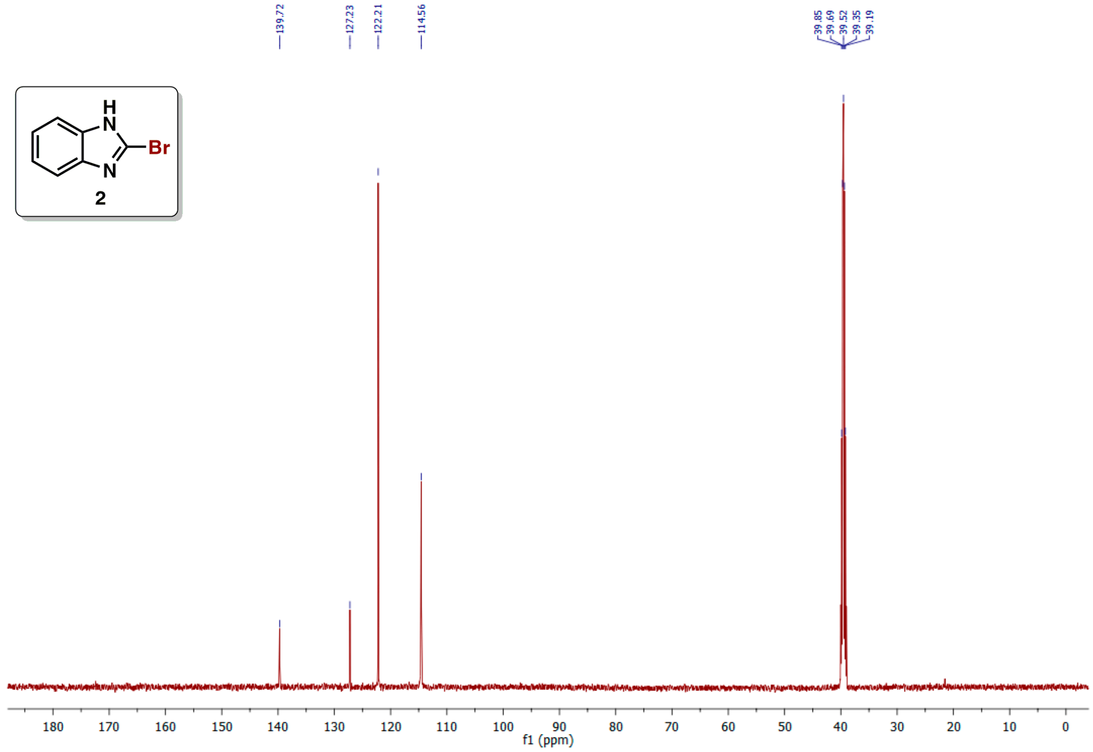

FTMS spectrum of **2** (ESI-TOF)

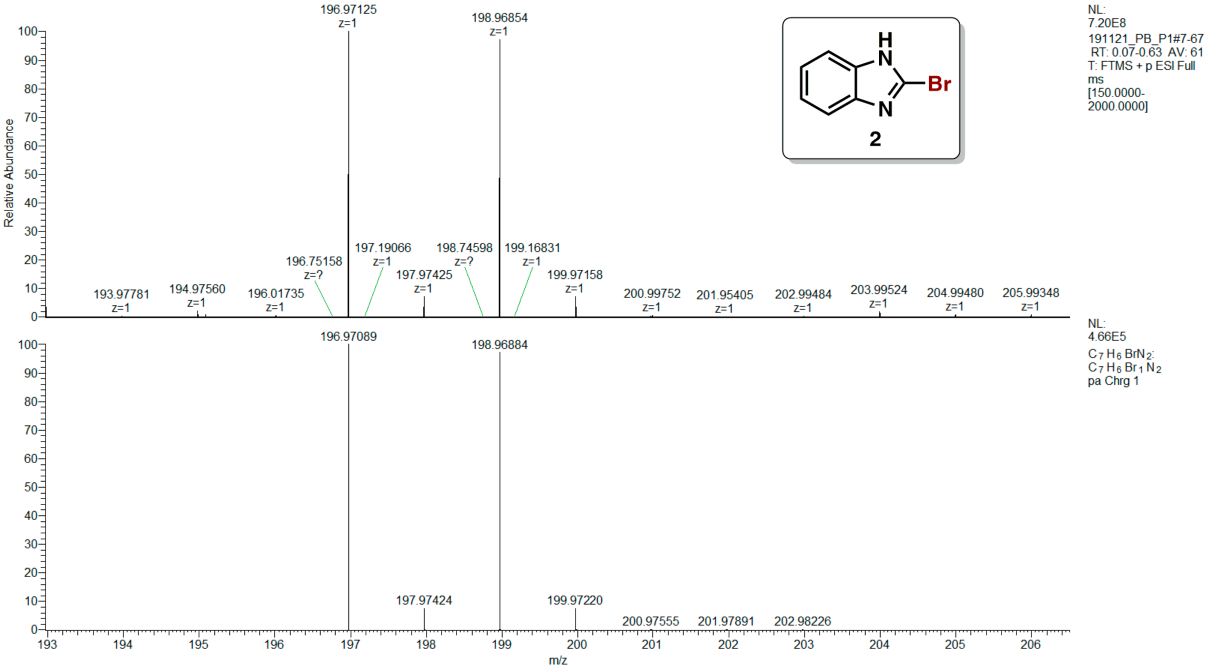

2,4,5,6,7-Pentabromo-1H-benzimidazole (3)

^{1}H NMR spectrum of **3** (500 MHz, DMSO-d_6)

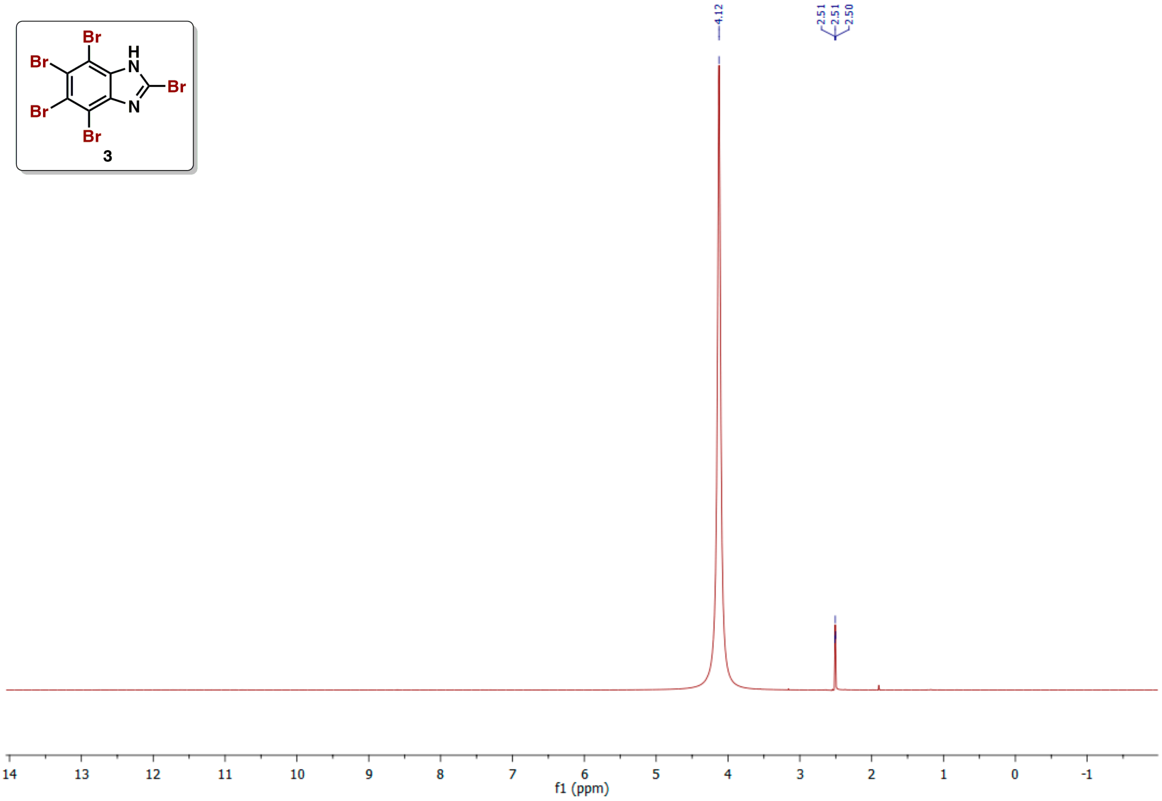

^{13}C NMR spectrum of **3** (126 MHz, DMSO-d_6 + 2 drops of 2M HCl_{aq})

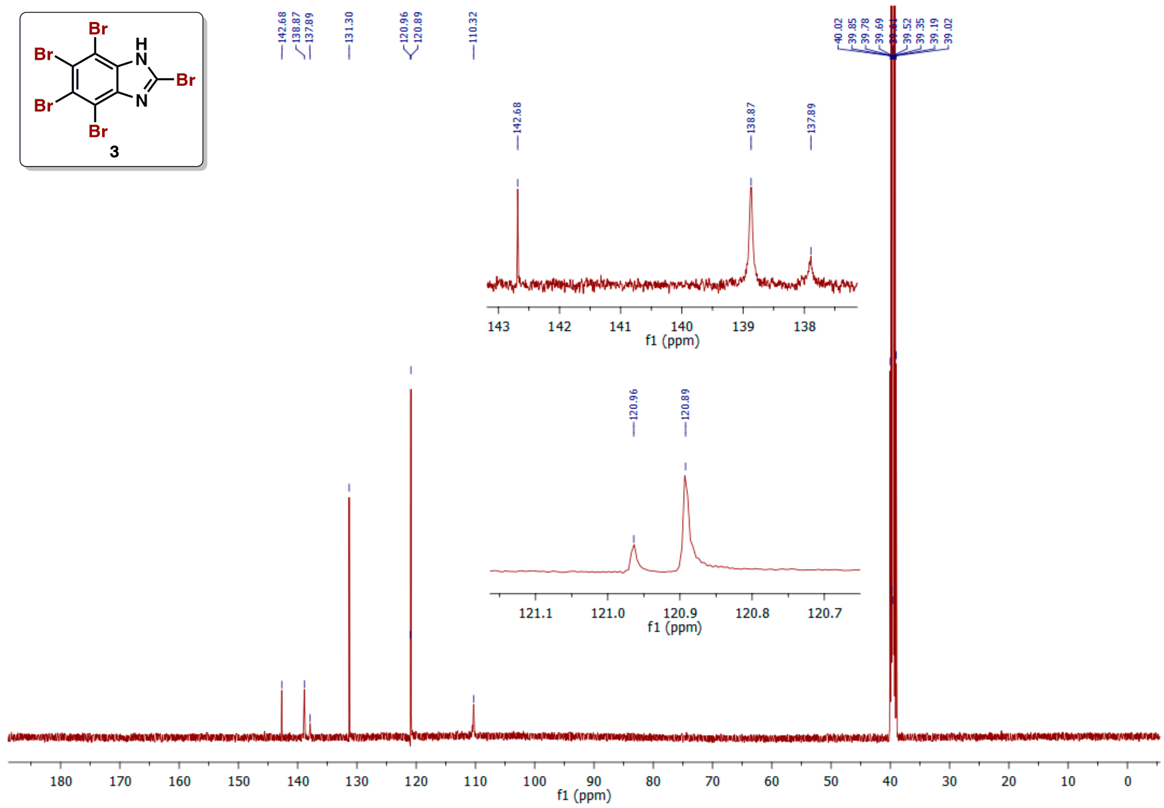

FTMS spectrum of **3** (ESI-TOF)

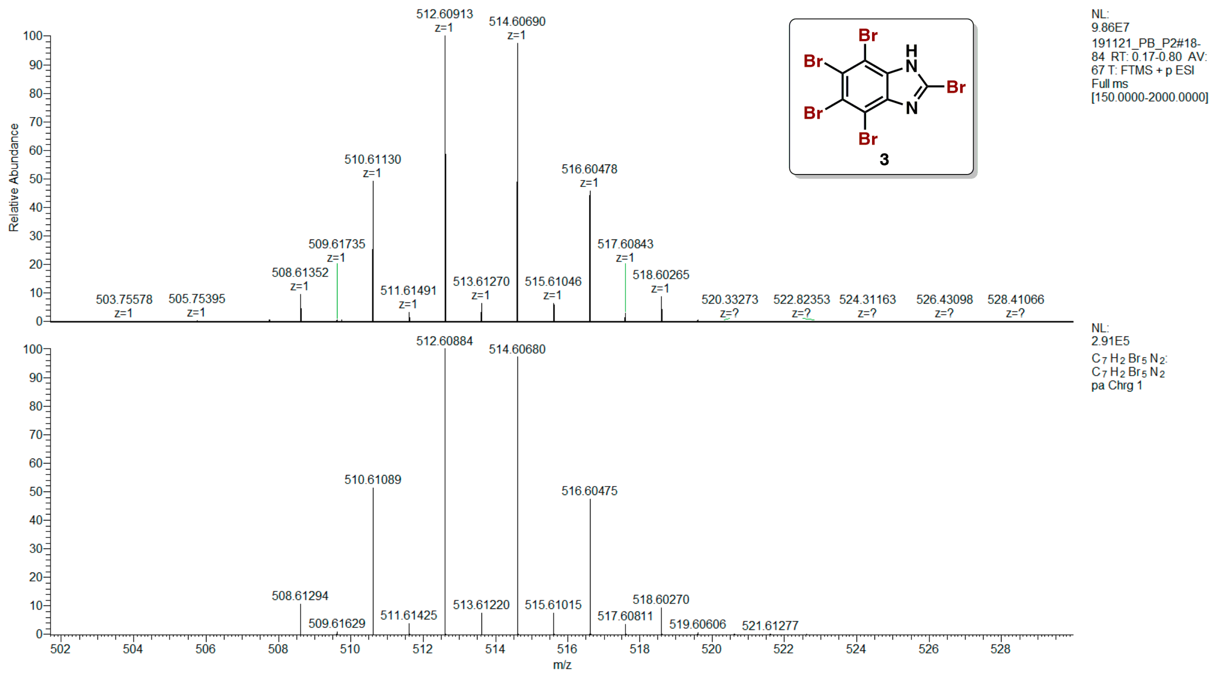

4,5,6,7-Tetrabromo-N,N-dimethyl-1H-benzimidazol-2-amine **(DMAT, 4)**

^{1}H NMR spectrum of **4** (500 MHz, DMSO-*d*$_6$)

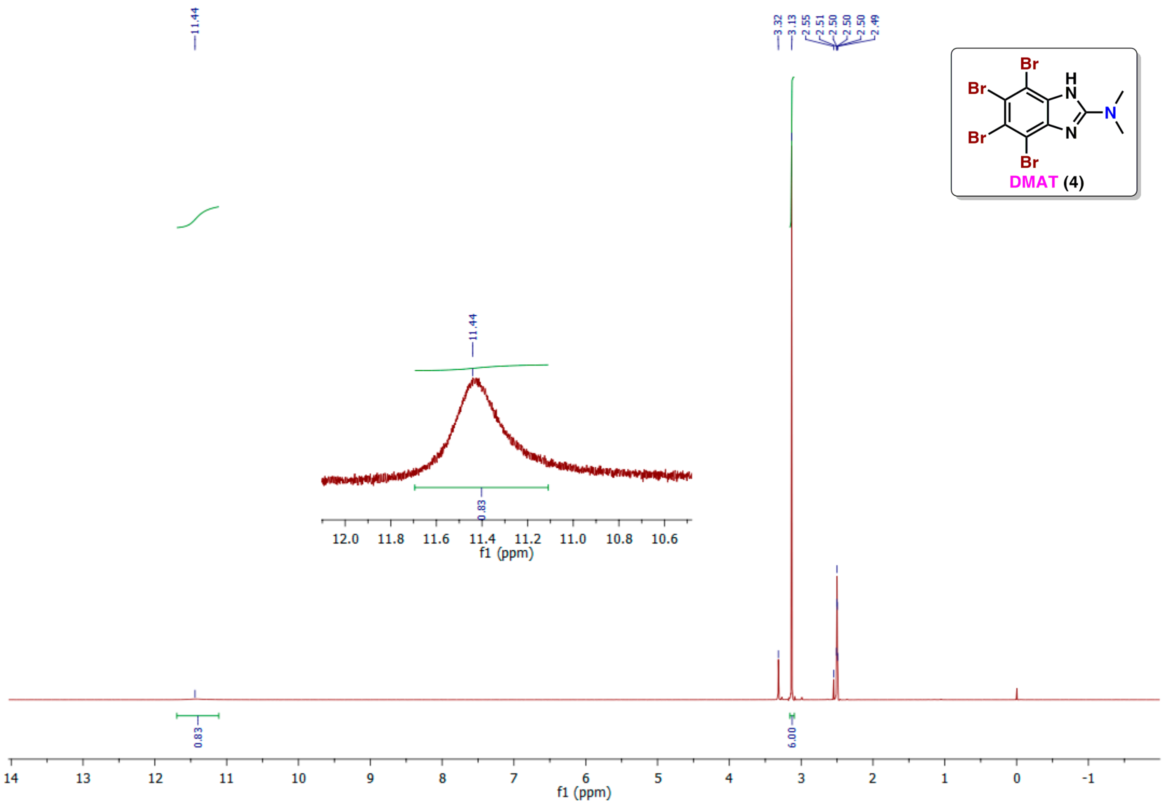

^{13}C NMR spectrum of **4** (126 MHz, DMSO-*d*$_6$)

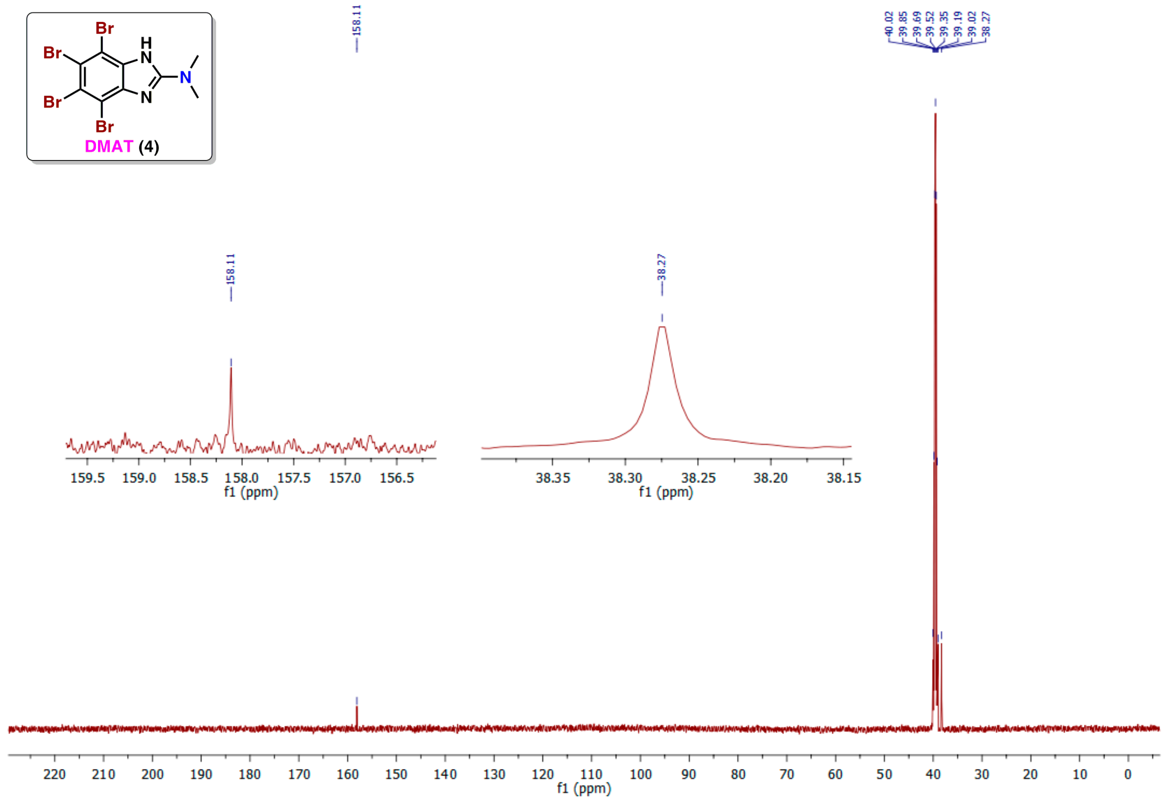

FTMS spectrum of **4** (ESI-TOF)

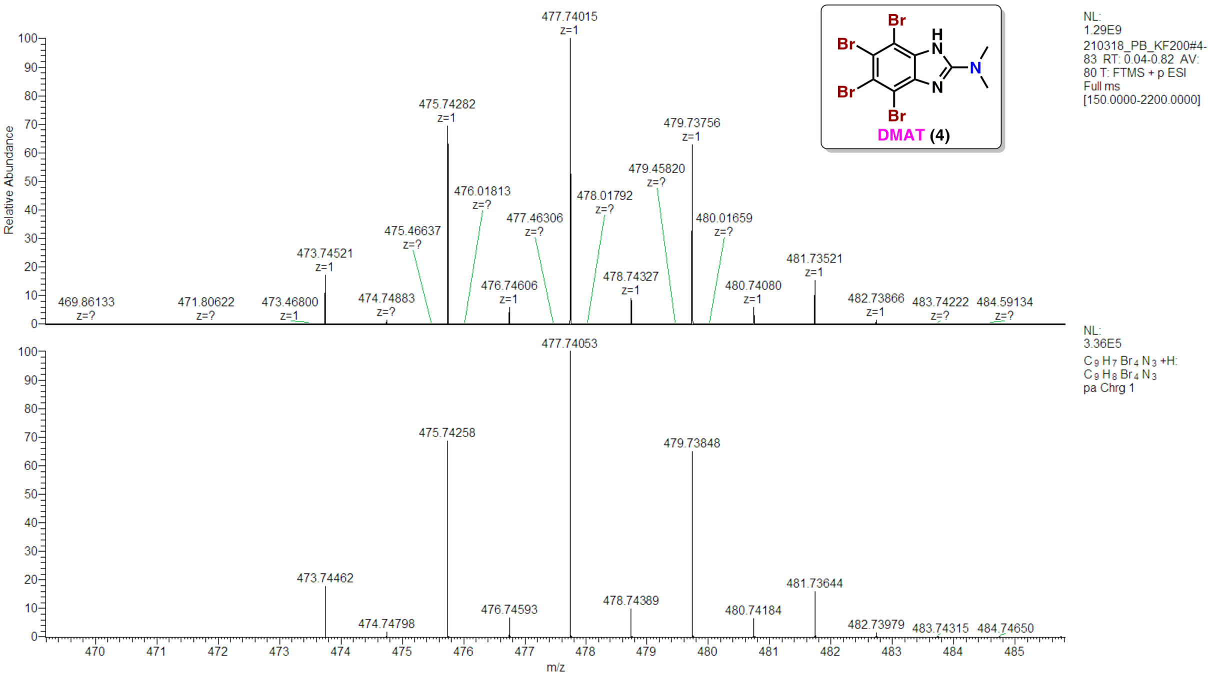

1-[(4,5,6,7-Tetrabromo-1H-benzimidazol-2-yl)amino]propan-2-ol (*rac*-**5**)

^{1}H NMR spectrum of *rac*-**5** (500 MHz, DMSO-d_6)

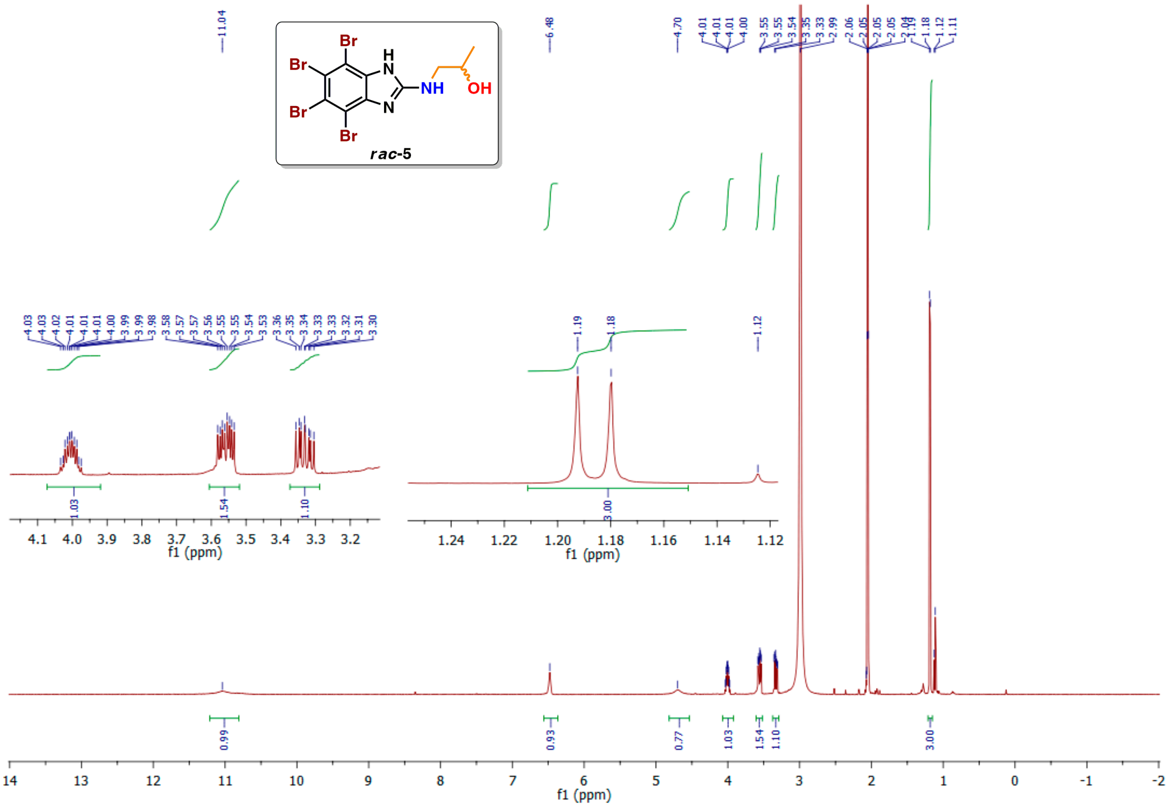

^{1}H NMR spectrum of *rac*-**5** (500 MHz, DMSO-d_6)

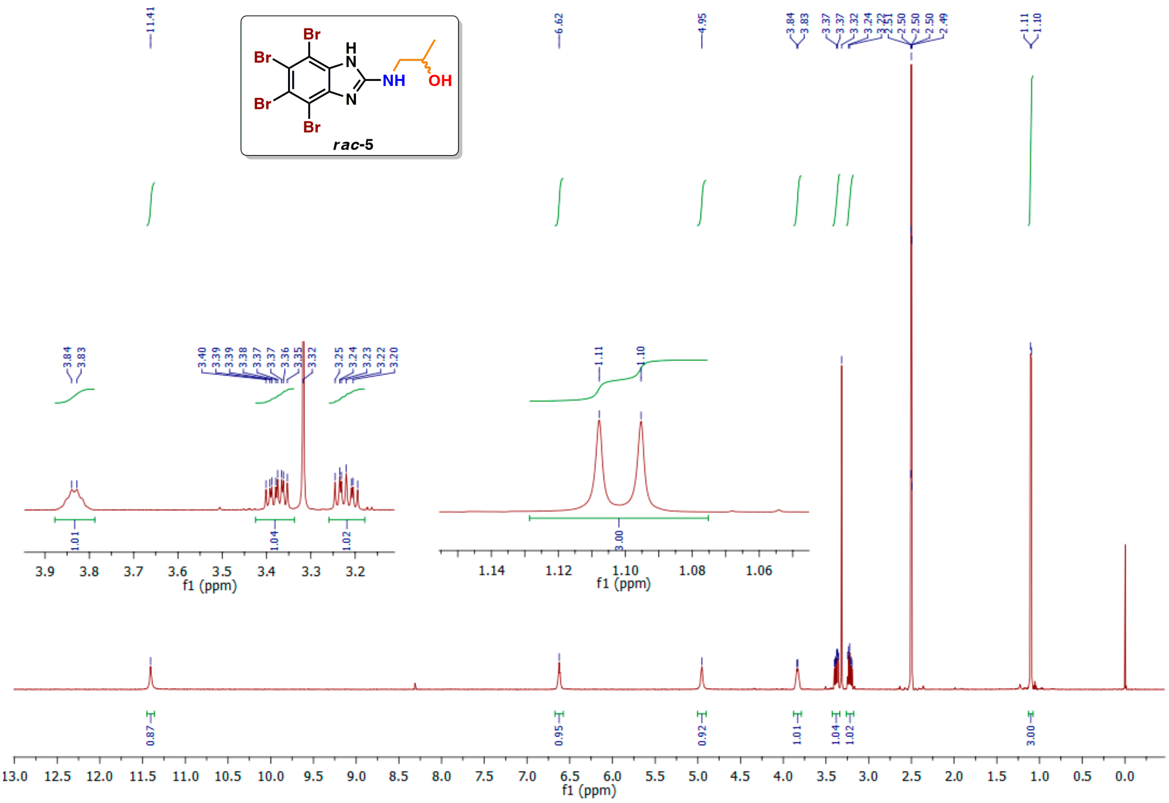

^{13}C NMR spectrum of *rac*-**5** (126 MHz, DMSO-d_6 + 2 drops of 2M HCl_{aq})

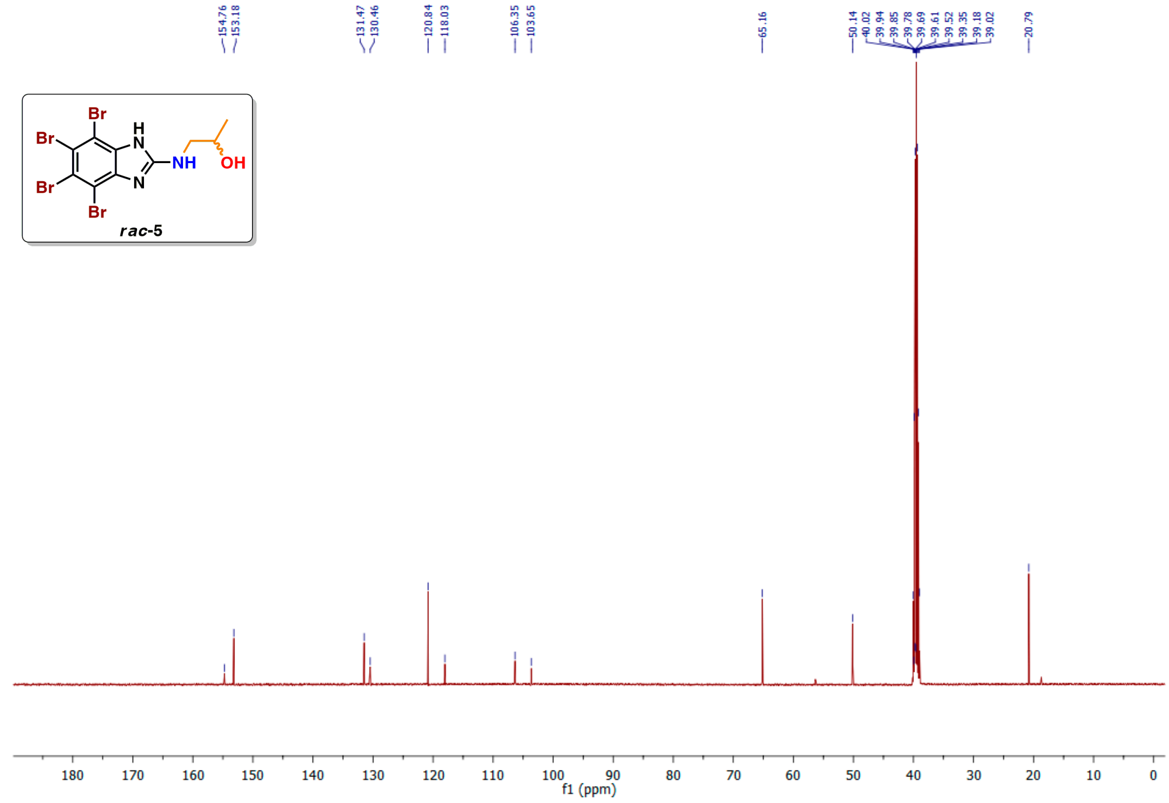

FTMS spectrum of *rac*-**5** (ESI-TOF)

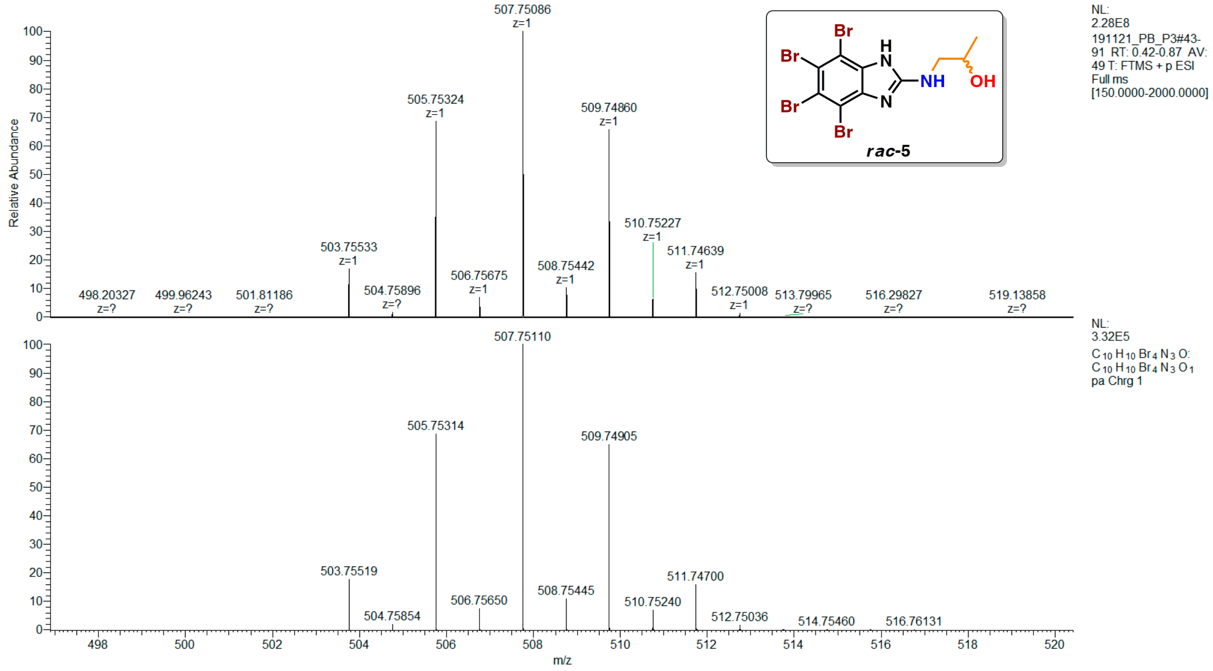

1,1,1-Trifluoro-3-[(4,5,6,7-tetrabromo-1H-benzimidazol-2-yl)amino]propan-2-ol (rac-6)

^{1}H NMR spectrum of *rac*-**6** (500 MHz, DMSO-d_6)

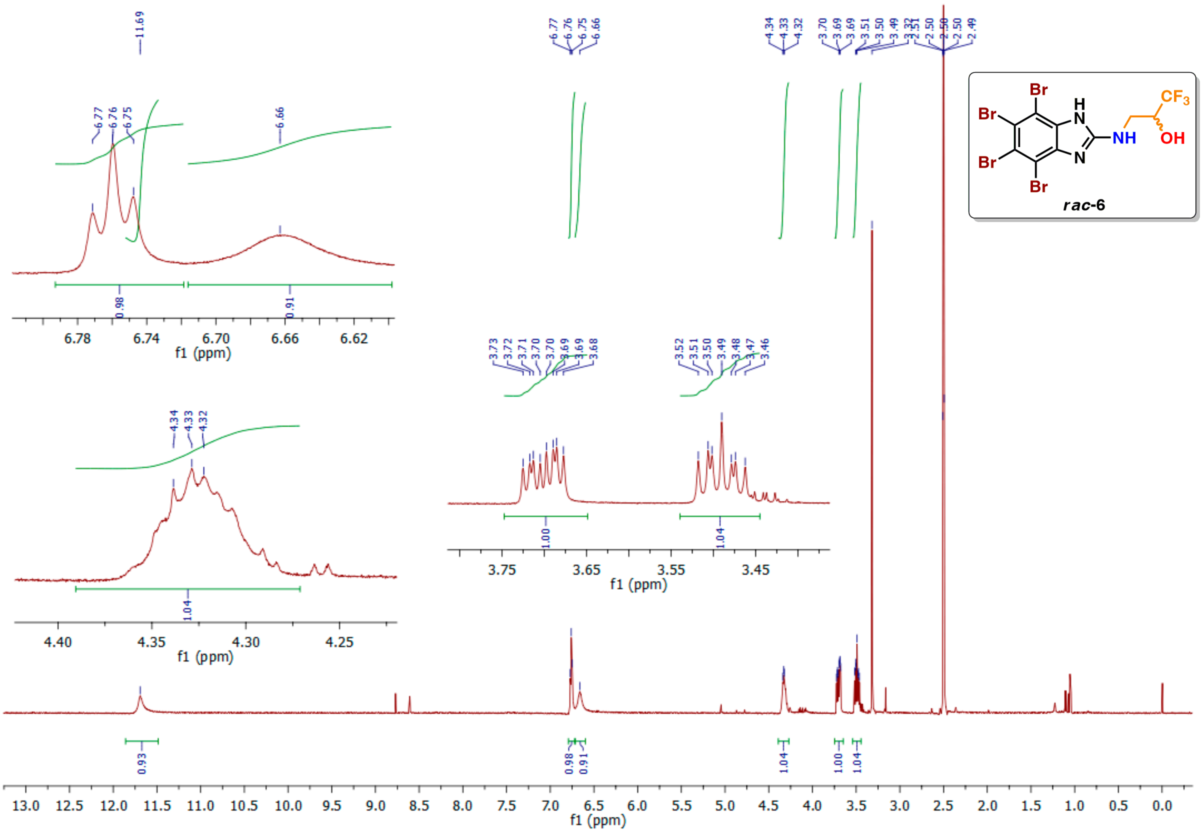

^{19}F NMR spectrum of *rac*-**6** (470 MHz, CD_3CN)

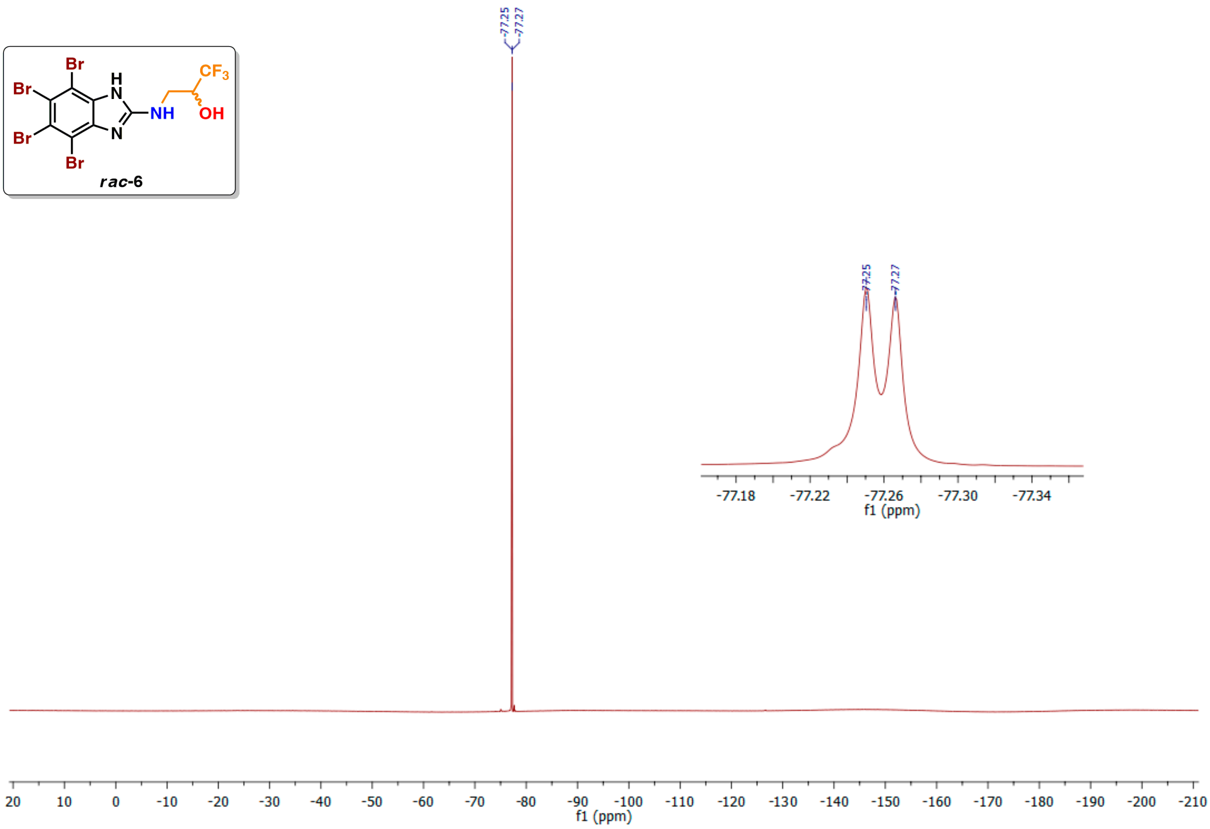

FTMS spectrum of *rac*-**6** (ESI-TOF)

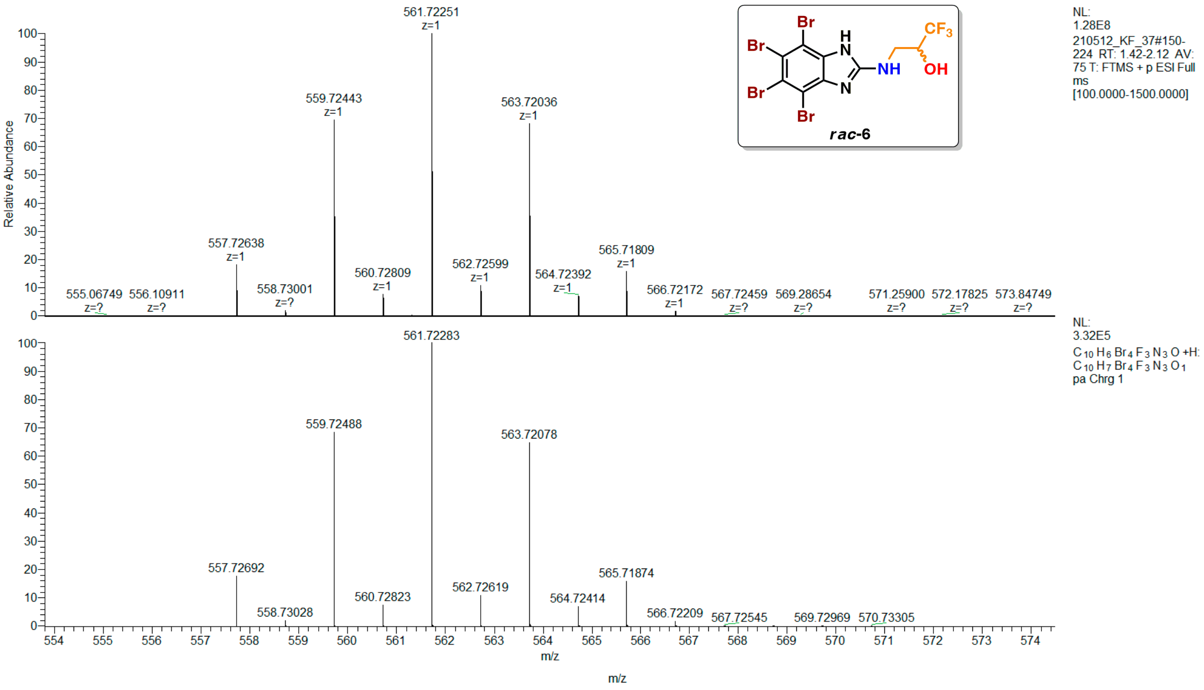

3-[(4,5,6,7-Tetrabromo-1H-benzimidazol-2-yl)amino]propan-1-ol (**7**)

^{1}H NMR spectrum of **7** (500 MHz, DMSO-d_6)

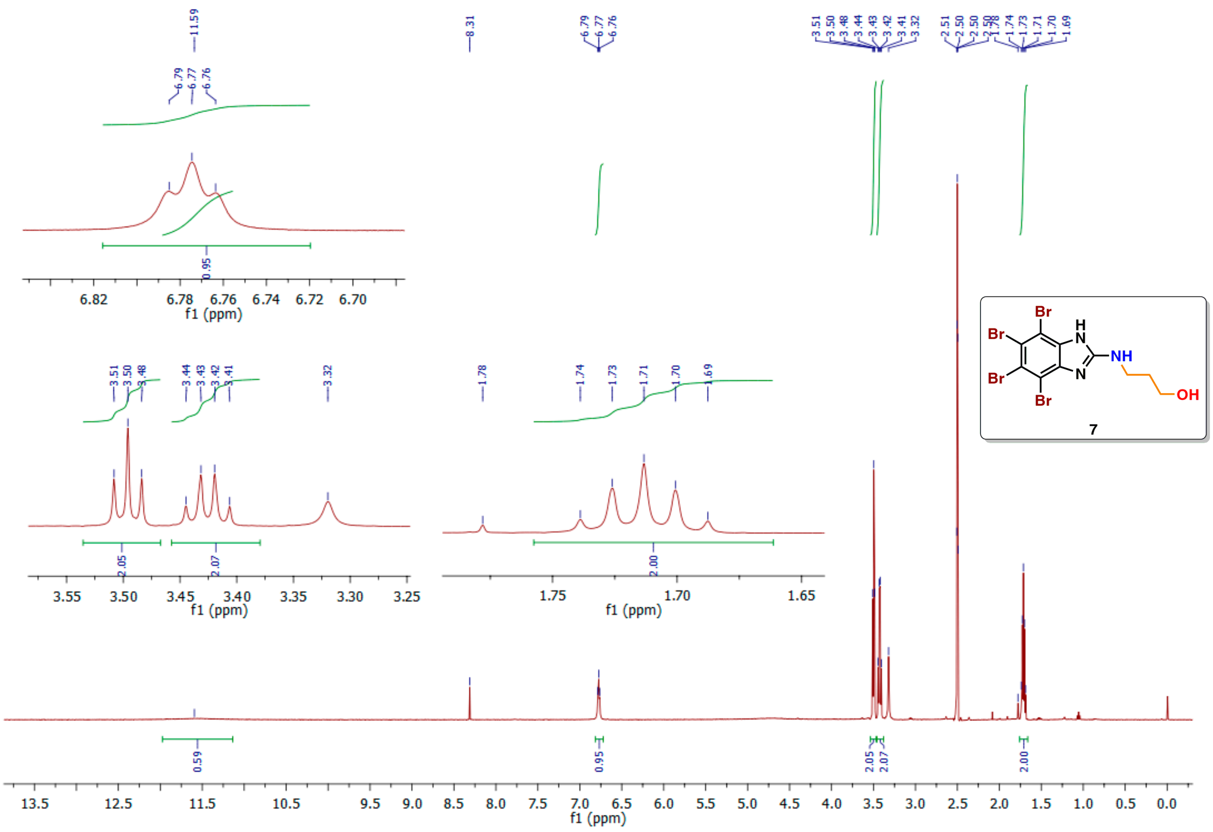

FTMS spectrum of **7** (ESI-TOF)

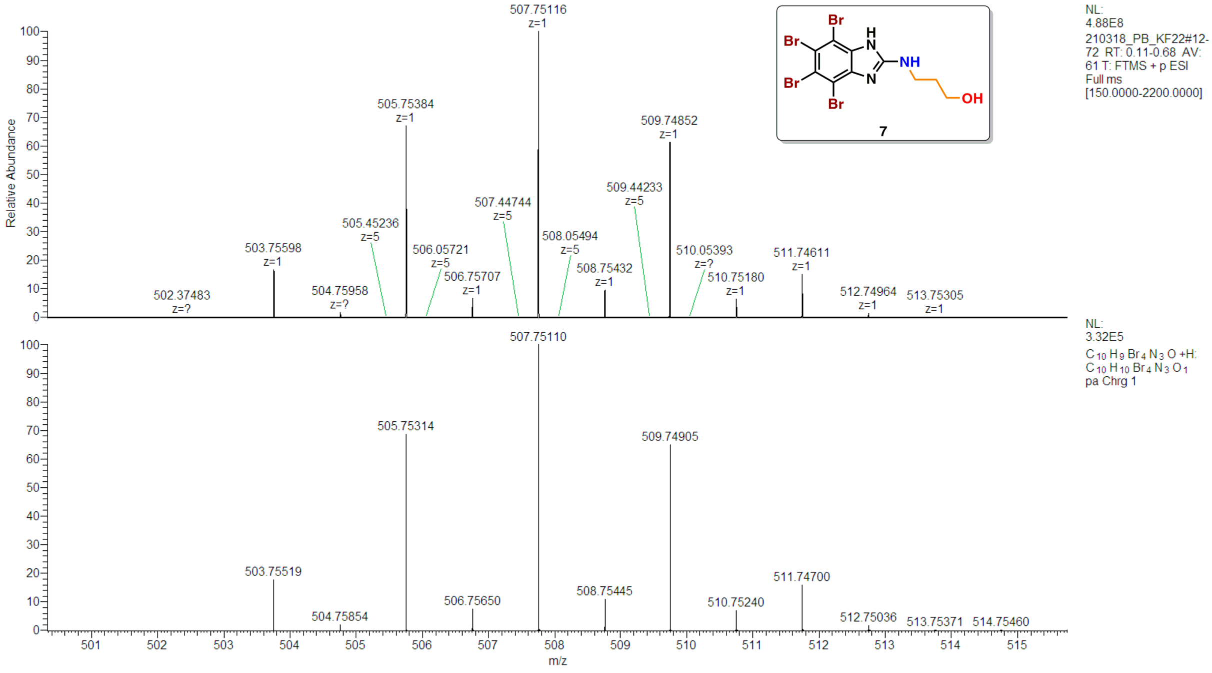

2-[Methyl(4,5,6,7-tetrabromo-1H-benzimidazol-2-yl)amino]ethanol (8)

^{1}H NMR spectrum of **8** (500 MHz, DMSO-d_6)

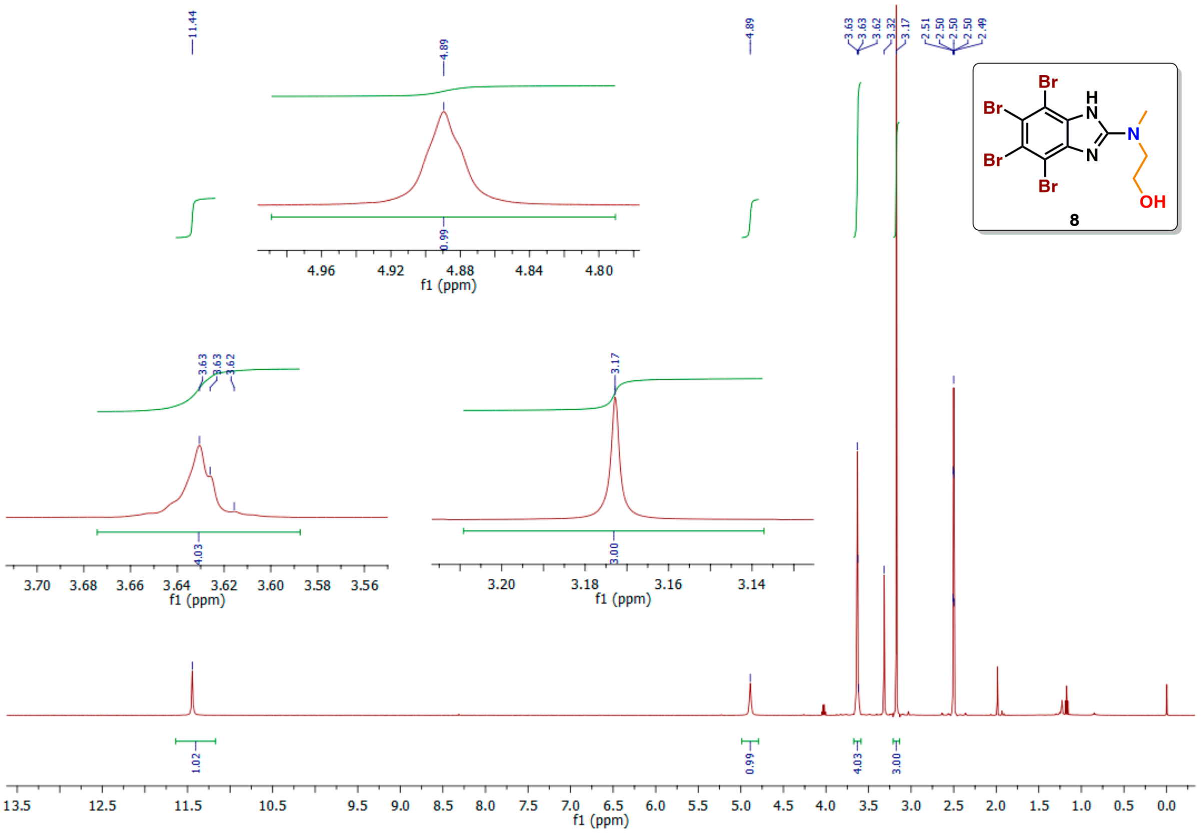

FTMS spectrum of **8** (ESI-TOF)

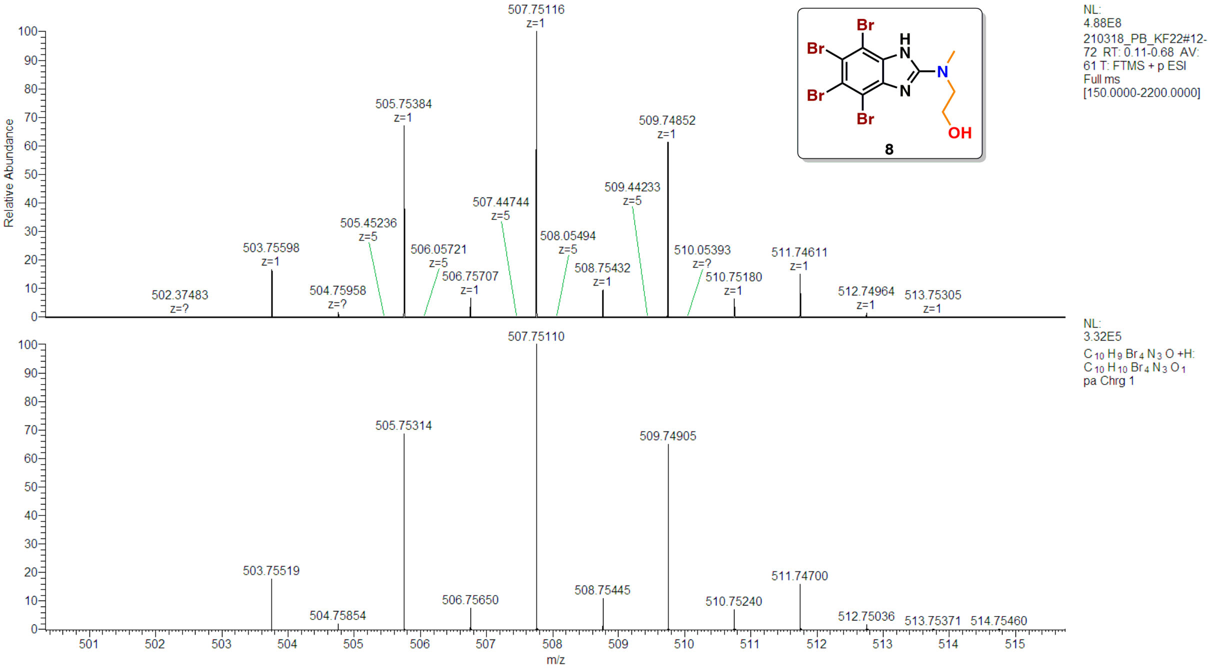

1-(Diethylamino)-3-[(4,5,6,7-tetrabromo-1H-benzimidazol-2-yl)amino]propan-2-ol (rac-9)

^{1}H NMR spectrum of *rac*-**9** (500 MHz, DMSO-d_6)

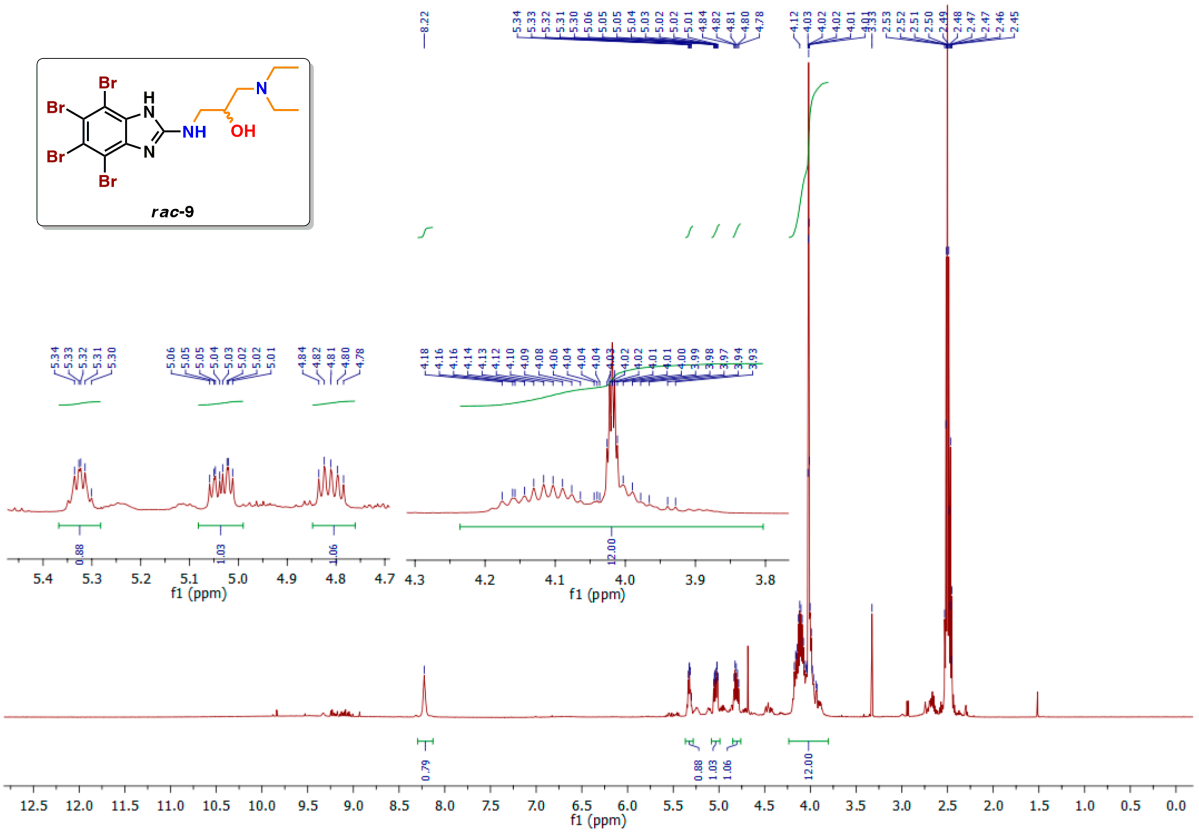

FTMS spectrum of *rac*-**9** (ESI-TOF)

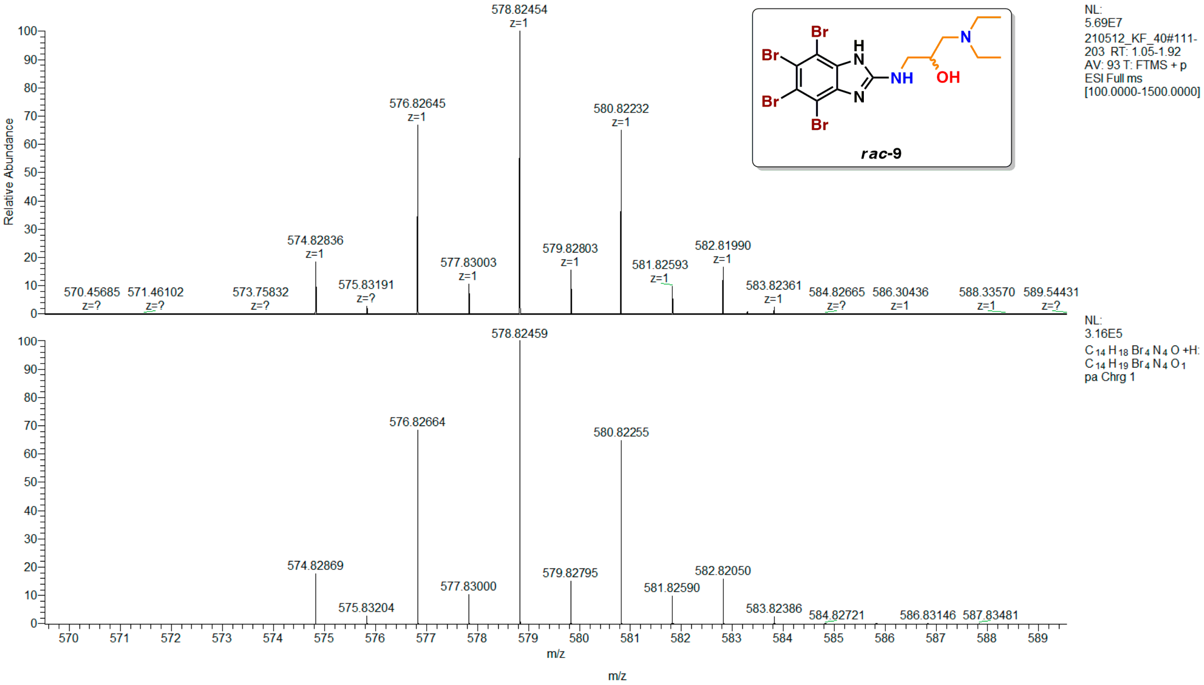

2-[(4,5,6,7-Tetrabromo-1H-benzimidazol-2-yl)amino]propane-1,3-diol (10)

^{1}H NMR spectrum of **10** (500 MHz, DMSO-d_6)

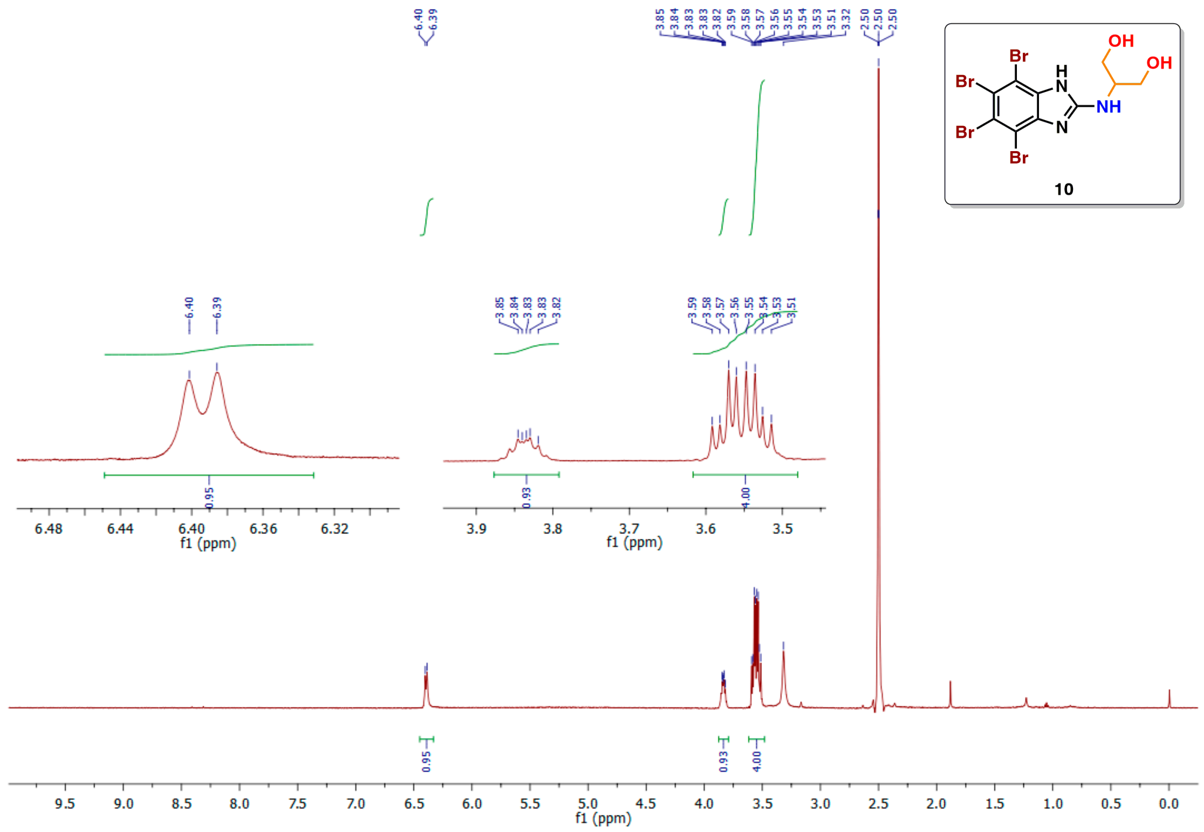

FTMS spectrum of **10** (ESI-TOF)

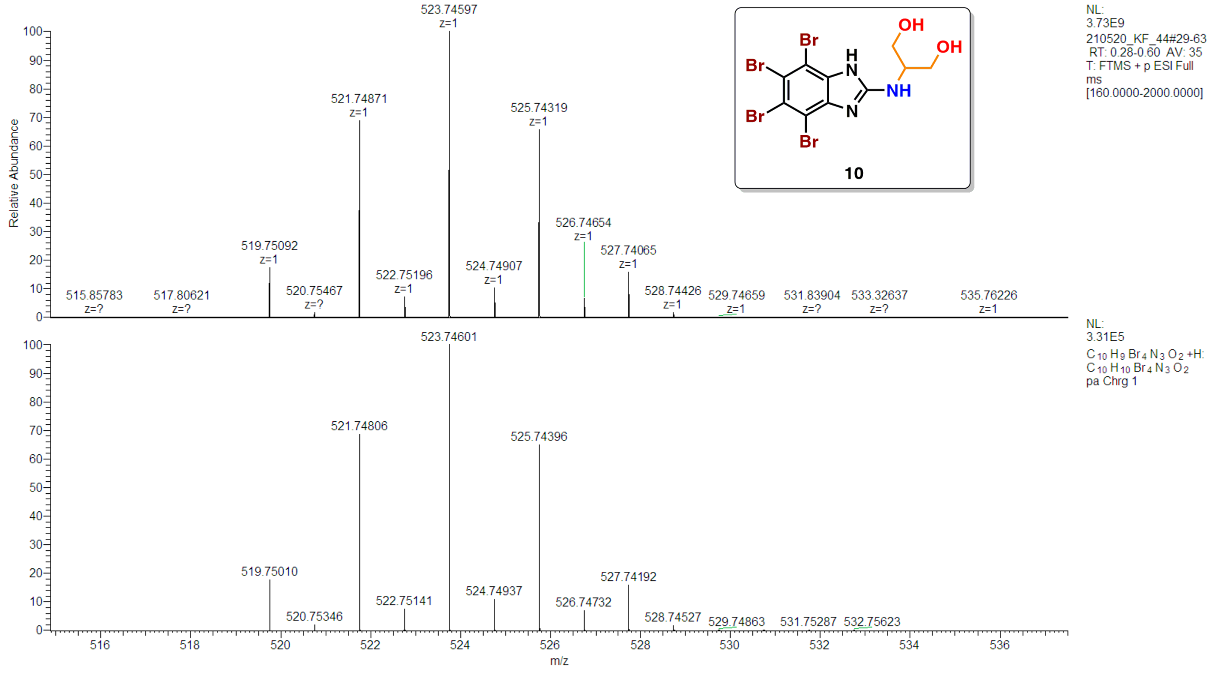

3-[(4,5,6,7-Tetrabromo-1H-benzimidazol-2-yl)amino]propane-1,2-diol (rac-11)

^{1}H NMR spectrum of *rac*-**11** (500 MHz, DMSO-d_6)

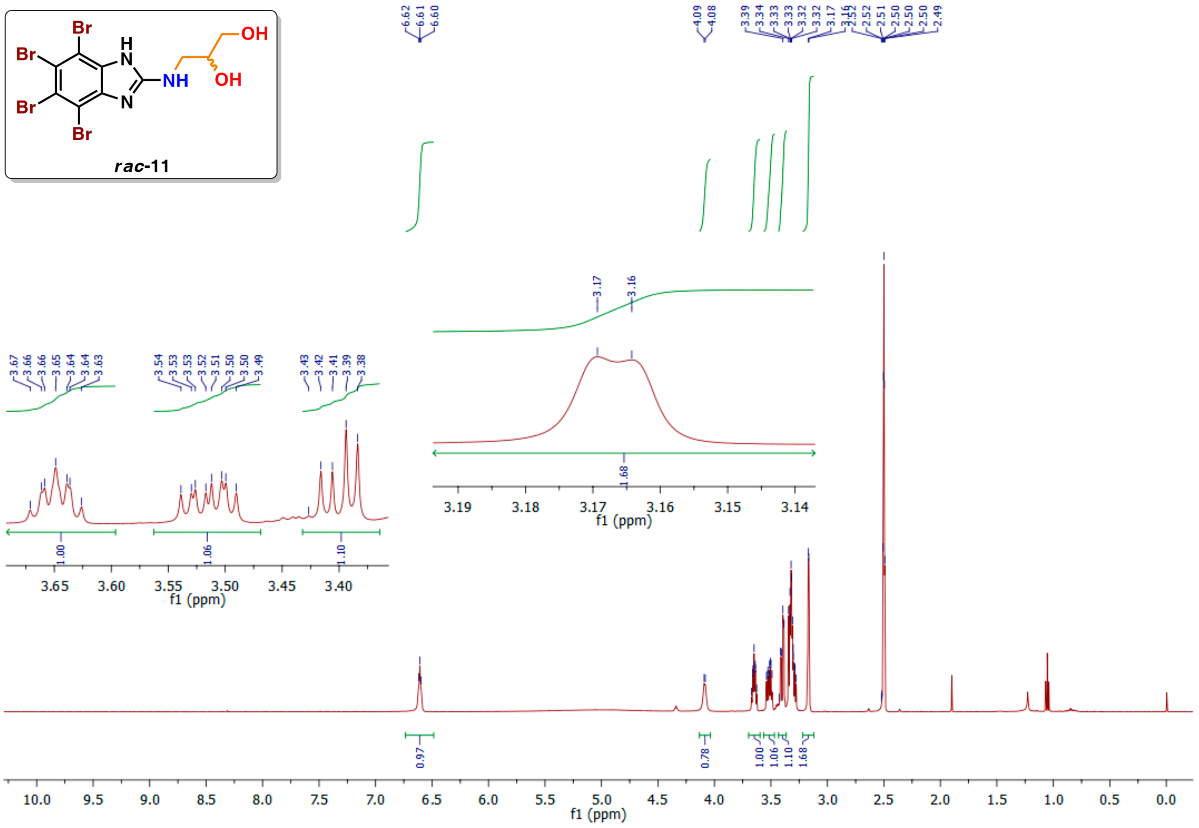

FTMS spectrum of *rac*-**11** (ESI-TOF)

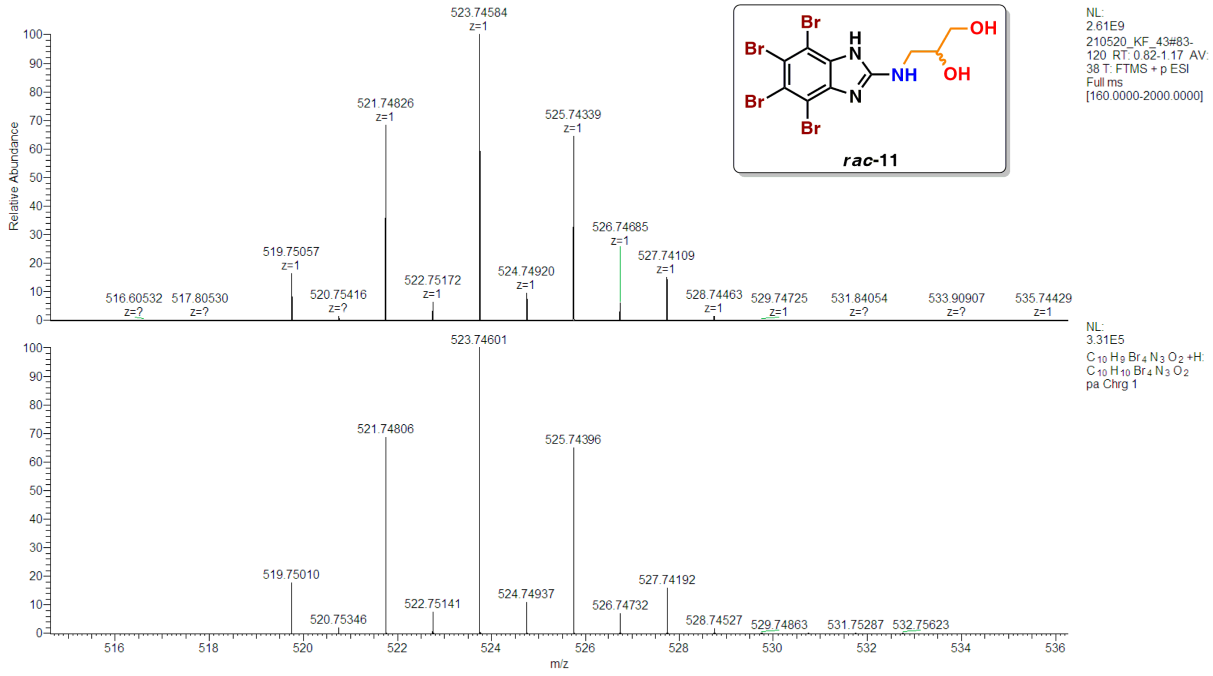

8. References.

1. Ellingboe, J.W.; Spinelli, W.; Winkley, M.W.; Nguyen, T.T.; Parsons, R.W.; Moubarak, I.F.; Kitzen, J.M.; Von Engen, D.; Bagli, J.F. Class III antiarrhythmic activity of novel substituted 4-[(methylsulfonyl)amino]benzamides and sulfonamides. *J. Med. Chem.* **1992**, *35*, 705–716, doi:10.1021/jm00082a011.
2. Andrzejewska, M.; Pagano, M.A.; Meggio, F.; Brunati, A.M.; Kazimierczuk, Z. Polyhalogenobenzimidazoles: synthesis and their inhibitory activity against casein kinases. *Bioorg. Med. Chem.* **2003**, *11*, 3997–4002, doi:10.1016/s0968-0896(03)00403-6.
3. Pagano, M.A.; Andrzejewska, M.; Ruzzene, M.; Sarno, S.; Cesaro, L.; Bain, J.; Elliott, M.; Meggio, F.; Kazimierczuk, Z.; Pinna, L.A. Optimization of protein kinase CK2 inhibitors derived from 4,5,6,7-tetrabromobenzimidazole. *J. Med. Chem.* **2004**, *47*, 6239–6247, doi:10.1021/jm049854a.
4. Daina, A.; Blatter, M.-C.; Baillie Gerritsen, V.; Palagi, P. M.; Marek, D.; Xenarios, I.; Schwede, T.; Michielin, O.; Zoete, V. Drug Design Workshop: A Web-Based Educational Tool to Introduce Computer-Aided Drug Design to the General Public. *J. Chem. Educ.* **2017**, *94*, 335–344, doi:10.1021/acs.jchemed.6b00596.
5. Trott, O.; Olson, A.J. AutoDock Vina: improving the speed and accuracy of docking with a new scoring function, efficient optimization, and multithreading. *J. Comput. Chem.* **2010**, *31*, 455–461, doi:10.1002/jcc.21334.
6. Wang, J.; Wolf, R.M.; Caldwell, J.W.; Kollman, P.A.; Case, D.A. Development and testing of a general amber force field. *J. Comput. Chem.* **2004**, *25*, 1157–1174, doi:10.1002/jcc.20035.
7. Cozza, G.; Girardi, C.; Ranchio, A.; Lolli, G.; Sarno, S.; Orzeszko, A.; Kazimierczuk, Z.; Battistutta, R.; Ruzzene, M.; Pinna, L.A. Cell-permeable dual inhibitors of protein kinases CK2 and PIM-1: structural features and pharmacological potential. *Cell. Mol. Life Sci.: CMLS* **2014**, *71*, 3173−3185, doi:10.1007/s00018-013-1552-5.
8. Dakin, L.A.; Block, M.H.; Chen, H.; Code, E.; Dowling, J.E.; Feng, X.; Ferguson, A.D.; Green, I.; Hird, A.W.; Howard, T., et al. Discovery of novel benzylidene-1,3-thiazolidine-2,4-diones as potent and selective inhibitors of the PIM-1, PIM-2, and PIM-3 protein kinases. *Bioorg. Med. Chem. Lett.* **2012**, *22*, 4599−4604, doi:10.1016/j.bmcl.2012.05.098.
9. Pettersen, E.F.; Goddard, T.D.; Huang, C.C.; Couch, G.S.; Greenblatt, D.M.; Meng, E.C.; Ferrin, T.E. UCSF Chimera--a visualization system for exploratory research and analysis. *J. Comput. Chem.* **2004**, *25*, 1605−1612, doi:10.1002/jcc.20084.
